# Understanding *Pseudomonas aeruginosa*–Host Interactions: The Ongoing Quest for an Efficacious Vaccine

**DOI:** 10.3390/cells9122617

**Published:** 2020-12-05

**Authors:** Maite Sainz-Mejías, Irene Jurado-Martín, Siobhán McClean

**Affiliations:** School of Biomolecular and Biomedical Sciences, University College Dublin, Belfield, Dublin 4, D04 V1W8, Ireland; maite.sainzmejias@ucd.ie (M.S.-M.); irene.juradomartin@ucd.ie (I.J.-M.)

**Keywords:** *Pseudomonas aeruginosa*, ESKAPE, host-pathogen interactions, virulence factors, immune response, vaccine antigens, adjuvants

## Abstract

*Pseudomonas aeruginosa* is a leading cause of chronic respiratory infections in people with cystic fibrosis (CF), bronchiectasis or chronic obstructive pulmonary disease (COPD), and acute infections in immunocompromised individuals. The adaptability of this opportunistic pathogen has hampered the development of antimicrobial therapies, and consequently, it remains a major threat to public health. Due to its antimicrobial resistance, vaccines represent an alternative strategy to tackle the pathogen, yet despite over 50 years of research on anti-*Pseudomonas* vaccines, no vaccine has been licensed. Nevertheless, there have been many advances in this field, including a better understanding of the host immune response and the biology of *P. aeruginosa*. Multiple antigens and adjuvants have been investigated with varying results. Although the most effective protective response remains to be established, it is clear that a polarised Th2 response is sub-optimal, and a mixed Th1/Th2 or Th1/Th17 response appears beneficial. This comprehensive review collates the current understanding of the complexities of *P. aeruginosa*-host interactions and its implication in vaccine design, with a view to understanding the current state of *Pseudomonal* vaccine development and the direction of future efforts. It highlights the importance of the incorporation of appropriate adjuvants to the protective antigen to yield optimal protection.

## 1. Introduction

*Pseudomonas aeruginosa* is a motile and aerobic Gram-negative bacillus, with great diversity and adaptability in a wide range of environments, including non-clinical (soil, aquatic environments or plants) and clinical settings (nosocomial infections and medical equipment, such as inhalers, respirators, and vaporisers) [1]. As an opportunistic pathogen, it belongs to the multi-drug resistant (MDR) ESKAPE pathogens, comprising *Enterococcus faecium, Staphylococcus aureus, Klebsiella pneumoniae, Acinetobacter baumannii*, and *Enterobacter* [2,3]. In 2017, MDR *P. aeruginosa* caused 32,600 infections among hospitalised patients and 2700 estimated deaths in the United States [4,5]. A broad spectrum of drugs is available for *P. aeruginosa* infections; however, the pathogen quickly develops tolerance to these agents due to its intrinsic resistome [6]. In 2017, carbapenem-resistant *P. aeruginosa* was included by the World Health Organisation (WHO) among the “critical” group of pathogens for which new antibiotics are urgently needed [7,8].

*P. aeruginosa* is responsible for opportunistic infections in immunocompromised individuals and patients with malignant diseases or HIV infection [9]. Moreover, chronic airway infections with *P. aeruginosa* are a significant co-morbidity in patients with cystic fibrosis (CF), bronchiectasis, chronic obstructive pulmonary disease (COPD) or ventilator-associated pneumonia (VAP) [3,10]. According to the annual report of the American Cystic Fibrosis Foundation, in 2018, 45.3% of CF patients from the United States were colonised with *P. aeruginosa* [11]. *P. aeruginosa* is a major threat due to its propensity to adapt and acquire resistance to antibiotics. Consequently, vaccines have the potential to be more effective interventions for the prevention and treatment of *Pseudomonas* infections. Optimal vaccine design must consider complex host–pathogen interactions to identify effective antigens and delivery systems. Hence, this review describes the host immune response against *P. aeruginosa*; the factors that allow the adaptation of the pathogen to the host; and the numerous vaccine candidates and adjuvants that have been evaluated over half a century of *Pseudomonas* vaccine development.

## 2. *P. aeruginosa* Virulence Factors and Adaptation to the Lung Environment

**Figure 1:**
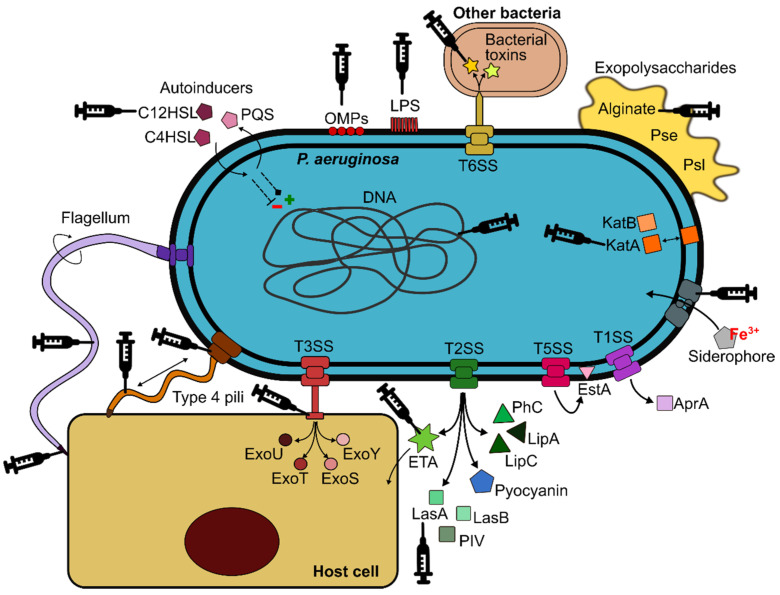
The main *P. aeruginosa* virulence factors involved in pathogenesis during pulmonary infections. The components highlighted with syringes have already been evaluated as vaccine antigens.

*P. aeruginosa* possesses a wide arsenal of virulence factors that contribute to its pathogenicity (Figure 1). Lipopolysaccharide (LPS) is the major structural component and protective element of the external leaflet in the outer membrane (OM). It causes tissue damage due to the endotoxic properties of the lipid A, mediates interactions with host receptors, and may play an indirect role in host-cell attachment [12,13]. It also influences resistance to antibiotics and the formation of outer membrane vesicles (OMVs) and biofilms [14]. The OM also contains a range of proteins (OMPs) involved in numerous functions, such as the exchange of nutrients, antibiotic resistance or adhesion, although most of them remain unknown [15]. The single polar flagellum of *P. aeruginosa* is essential for colonisation of the host. It is primarily responsible for swimming and swarming, being closely linked to chemotactic signalling [16]. It also participates in bacterial adhesion to host surfaces via mucin MUC1 and Lewis x glycotypes [17]. Type IV pili (T4P) are polarly located retractile appendages, crucial for the initiation of infection by controlling twitching motility and attachment to host cells [18]. The flagellum, T4P, and other adhesins are key factors for the formation of robust *P. aeruginosa* biofilms, which represent major challenges for treatment due to their high resistance to antibiotics, disinfectants and the host immune response [19,20]. Exopolysaccharides (alginate, Psl and Pel) also contribute to the biofilm matrix, impairing bacterial clearance, and promoting the establishment of chronic and highly recalcitrant infections [19,21].

*P. aeruginosa* uses five secretory systems (I, II, III, V and VI) to release a wide variety of toxins and hydrolytic enzymes that attack the host to both the intracellular and extracellular milieu [22,23]. The Type III secretion system (T3SS) is critical for the destruction of host defences through the injection of four cytotoxic effectors (ExoU, ExoT, ExoS and ExoY) [24]. Exotoxin A (ETA) is the most toxic product released by *P. aeruginosa*, inhibiting host protein synthesis due to its ADP-ribosylating activity, ultimately leading to irreversible cell death [25]. Pyocyanin also has toxic effects, associated with disease severity and decline in lung function [26]. In addition, several proteolytic (LasA and LasB elastases, alkaline protease (AprA) or type IV protease (PIV)) and lipolytic (LipA and LipC lipases, phospholipase C (PhC) enzymes or esterase A (EstA)) enzymes are also produced, playing important roles during acute infection and controlling other virulence factors [27]. The production of rhamnolipids by *P. aeruginosa* further contributes to the disruption of the respiratory epithelium by degrading lung surfactant and disrupting tight junction integrity [28,29,30]. Moreover, a number of antioxidant enzymes that help *P. aeruginosa* overcome oxidative stress in the host are also expressed, including catalases (KatA, KatB, and KatE), alkyl hydroperoxide reductases and superoxide dismutases [31]. Production of siderophores (pyoverdine and pyochelin) or other iron uptake systems is also critical for *P. aeruginosa* survival in this environment, where this essential nutrient is scarce [32,33].

The regulation of all these virulence factors is cell density-dependent via the release of autoinducers of four quorum sensing (QS) systems (Las, Rhl, Pqs and Iqs). They are interconnected in a hierarchical manner, creating a highly adaptable network that responds to external stressors and provides *P. aeruginosa* with an extraordinary plasticity that facilitates successful colonisation of a broad range of niches [34]. This adaptability enables *P. aeruginosa* to persist in the respiratory tract of susceptible patients, thereby establishing chronic infections even for decades, especially in people with CF [35]. CF is an autosomal recessive disease caused by mutations in the CF transmembrane conductance regulator (CFTR) gene. CFTR deficiencies result in a dehydrated airway surface liquid (ASL) which facilitates bacterial colonisation of the airway [36]. *P. aeruginosa* is exposed to a diverse range of stressors in the CF lung, including osmotic, oxidative and nitrosative stresses, sublethal concentrations of antibiotics, and the presence of other microorganisms [37]. Hence, the CF lung is a heterogeneous, hostile, and stressful environment, which induces several evolutionary changes in *P. aeruginosa* populations. Thus, a multitude of genomic and phenotypic adaptations that promote bacterial survival by attenuating virulence and avoiding immune recognition have been reported [2,38]. These adaptations include: (i) emergence of hypermutators, (ii) appearance of morphology variants, such as small colony variant (SCV) and rugose small colony variant (RSCV), (iii) auxotrophy, (iv) overproduction of alginate and loss of flagellum and pili, leading to a sessile-biofilm lifestyle, (v) changes in the LPS (loss of *O*-antigen and structural modifications of lipid A), (vi) selection against T3SS and loss of cytotoxicity, (vii) reduction in communication systems (QS), (viii) change in iron uptake strategy from siderophores towards haemoglobin utilisation, (ix) acquisition of antibiotic resistance and (x) loss of virulence [2,39,40,41,42].

In addition to the environmental impact of CFTR mutations there is also variability between CF patients, despite identical CFTR genotypes, indicating that other genetic factors contribute to the severity of lung disease. This heterogeneity can be explained in part by the presence of modifier genes [43]. Interestingly, these genes also influence the course of *P. aeruginosa* infection. Emond et al. observed that variants in the DCTN4 gene were associated with the age of first *P. aeruginosa* airway infection, time to chronic *P. aeruginosa* infection and mucoid *P. aeruginosa* in individuals with CF [44]. Furthermore, another CF modifier gene, SLC6A14, affected the attachment of *P. aeruginosa* in both mice and humans, since it transports a bacterial attachment-promoting metabolite (l-arginine) out of the host ASL [45]. More recently, Castaldo et al. related the T2R38 genotype to the severity of sinonasal disease and the occurrence of *P. aeruginosa* pulmonary colonisation in 210 CF patients, suggesting that T2R38, which encodes a taste receptor, is a novel modifier gene in CF [46]. These and other modifier genes contribute to the diversity of CF disease, contributing to *P. aeruginosa* pathogenesis and the adaptation of the bacterium to the host environment.

## 3. Host Immune Response against *P. aeruginosa*

### 3.1. Recognition

**Figure 2:**
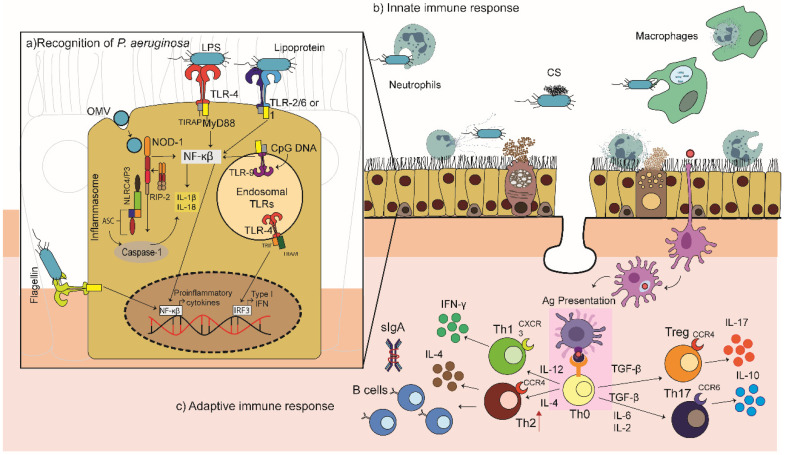
Host immune response against *P. aeruginosa* infections in the pseudostratified respiratory epithelium. (**a**) Recognition of *P. aeruginosa*. Pathogen recognition receptors (PRRs) located on the immune cells (TLR2, TLR4, TLR5, TLR9) recognise the PAMPs of *P. aeruginosa* (Lipoprotein, LPS, flagellin, and CpG-DNA, respectively) and trigger the production of pro-inflammatory cytokines and chemokines. OMV endocytosis activates the NF-κB pathway. (**b**) Innate immune response. (i) Neutrophils, recruited in response to *P. aeruginosa* (ii) Macrophages, which phagocytise bacteria or dying neutrophils (iii) complement system (CS). (**c**) Adaptive immune response. A skewed Th2 response occurs during *P. aeruginosa* infection with high but inefficient antibody production. In addition, the production of sIgA seems to be relevant, as its levels may correlate with the status of *Pseudomonas* infection.

*P. aeruginosa* expresses powerful agonists of Toll-like receptors (TLR), TLR2, TLR4, TLR5 and TLR9, which recognise lipopeptides, LPS, flagellin, and non-methylated bacterial CpG DNA, respectively (Figure 2) [47,48,49]. The TLR4-dependent inflammatory response to LPS, in particular, is considered vital for infection clearance [48]. The role of the TLR2 and TLR9 in the recognition of *P. aeruginosa* has also been explored. Initially, their involvement was thought to be weak and not very relevant [50,51]; however, in murine models of acute *P. aeruginosa* pneumonia, deficiencies in either TLR9 or TLR2 increased the resistance of mice to *P. aeruginosa* infections, which could be associated with their roles in the repression of inflammatory cytokines, i.e., the elimination of TLR2 or TLR9 may increase the capacity to rapidly clear bacteria [52,53]. TLR4 and TLR5 appear to be essential for the appropriate immune response against *P. aeruginosa* [49,54,55,56,57]. Blocking of TLR5 with anti-TLR5 antibodies (10 μg/mL) suppressed the ability of *P. aeruginosa*-infected lung cells to secrete invasive *P. aeruginosa* lung infection in mice [58]. The TLR4 senses LPS, leading to the activation of two distinct inflammatory cytokines (TNF-α, IL-6, and IL-8) [59], and its absence increases susceptibility to signalling pathways: (i) the primary response pathway of myeloid differentiation 88 (MyD88) and (ii) the adaptive pathway containing the Toll/IL-1R domain that induces beta interferon (IFN-β; TRIF pathway). MyD88 activates the nuclear factor kappa light chain enhancer of activated B cells (NF-κB), allowing the activation of a multitude of pro-inflammatory cytokines and chemokines (IL-6, TNF-α, and the macrophage inflammatory protein (MIP)-2). TRIF drives the transcription of chemokines IFN-α and IFN-β, RANTES (regulated in the activation of normal expressed and secreted T cell expressed) and IP-10 (Interferon γ-inducible protein 10) [60]. A TLR4/MD2 agonistic monoclonal antibody, UT12, promoted host defence against chronic *P. aeruginosa* lung infection in mice, increasing neutrophil levels and concentrations of inflammatory MIP-2 in the lungs and improving bacterial clearance [61].

The role of MyD88, an adaptor molecule for almost all TLRs, is especially important since several studies have shown that it is necessary for the rapid recruitment of neutrophils to the site of infection [62]. Blockage of multiple TLR pathways in mice (e.g., TLR2/TLR4/TLR5) did not compromise their response to *P. aeruginosa* as mice lacking MyD88 [63]. Hussain et al. recently demonstrated that the flagella receptor TLR5 is physically associated with the LPS receptor TLR4, diverting TLR4 signalling to the MyD88 pathway. After exposure of primary murine macrophages to ultra-pure LPS, TLR5 was co-immunopreserved with MyD88, TLR4 and LPS, suggesting an updated paradigm for TLR4/TLR5 signalling [64].

*P. aeruginosa* virulence factors target the host cell cytosol and activate the assembly of multi-molecular signalling platforms in immune cells known as inflammasomes. The role of NLRC4 and NLRP3 inflammasomes have been highlighted in the recognition and response to *P. aeruginosa* infections [65]. *P. aeruginosa* infection induces the assembly of the NLRP3 inflammasome and the successive secretion of caspase-1 and IL-1β in human macrophages. Interestingly, human cathelicidin LL-37/h-CAP18 may act as a second signal to promote the altruistic cell death of *P. aeruginosa*-infected epithelial cells, acting as a “fire alarm” to enhance the rapid escalation of protective inflammatory responses to uncontrolled infection by *P. aeruginosa*. The infected epithelial cells then release IL-1β and IL-18 promoting neutrophil influx [66].

In CF airway epithelial cells, *P. aeruginosa* infection enhanced mitochondrial Ca^2+^ uptake, leading to activation of the NLRP3 [67]. However, Huus et al. showed that *P. aeruginosa* isolates from CF patients failed to induce inflammatory activation, as measured by the secretion of IL-1β and IL-18 and pyroptotic cell death. It was suggested that the inflammasome evasion observed in CF patients may be responsible for the decreased expression of the *P. aeruginosa* virulence factors which activate the inflammasomes, such as reduced motility [68]. The NLRC4 inflammasome has been identified as an essential element in the innate response against *P. aeruginosa* activated mainly by flagellin and T3SS proteins. IL-1β secretion, in response to *P. aeruginosa*, is dependent on the NLRC4 inflammasome early in infection leading to the upregulation of other neutrophil chemoattractants, such as MIP-2 and KC/IL-8. This aids bacterial clearance in the early stages of infection but may lead to deleterious effects on the host during the later stages. IL-1β is elevated in the sputum and bronchoalveolar lavage fluid of CF patients colonised with *P. aeruginosa*, and antibiotic treatment in children was correlated with reduced production of IL-1β. However, the optimal level of IL-1β required for the elimination of bacteria by phagocyte recruitment without causing excessive host tissue damage is currently not yet well understood [49].

*P. aeruginosa* also activates Nod-like receptors (NLRs) by the release of OMVs, promoting TLR-dependent responses in epithelial cells through the delivery of proteins and LPS. The OMVs activate NF-κB signalling and mitogen-activated protein kinase (MAPK) in epithelial cells [49,69]. Importantly, the NLRs could be considered as potential therapeutic adjuvant targets capable of protecting lung damage during infection and could be the focal point for attenuating inflammatory responses in *P. aeruginosa* infected cells [49].

### 3.2. The Innate Immune Response

The recognition of *P. aeruginosa* pathogen-associated molecular patterns (PAMPs) elicits a potent inflammatory response, which is critical for the recruitment of neutrophils and macrophages, facilitating the bacterial clearance (Figure 2). However, an optimal host response is essential; a weak response with inefficient infiltration of phagocytic cells leads to unsuccessful bacterial killing and clearance, while an excessive response causes host tissue damage [49,70,71,72,73,74]. Massive recruitment of neutrophils into the infected respiratory tract is a hallmark of *P. aeruginosa* infection. In neutropenic mice, the lethal dose of *P. aeruginosa* was 100,000 times lower than that required by mice with normal neutrophil levels; observations which were replicated in rabbits and humans [71,75,76,77]. The factors that regulate the recruitment of neutrophils to *P. aeruginosa* infected sites have not been well characterised, but the recruitment is mediated, at least in part, by the production of ELR^+^ CXC chemokines, which are critical for neutrophil chemotaxis and activation [49,74,78,79]. The most relevant chemokine receptors on neutrophils are CXCR (CXC chemokine receptor) 1 and CXCR2. Mice express only the CXCR2 receptor, which binds to all ELR^+^ CXC chemokines, whereas humans also possess a more selective receptor, CXCR1, which binds specifically to the potent proinflammatory cytokine, IL-8 and to GCP-2 [78]. Both receptors are critical in the response to *P. aeruginosa* as they recruit neutrophils that aid in bacterial clearance and are well reviewed elsewhere [77,80]. The neutrophils act to kill *P. aeruginosa*, but can also contribute to host lung damage due to the production of reactive oxygen species (ROS) and the release of bactericidal proteins from their acidophilic granules [81]. Hence, an appropriate level of neutrophil recruitment is essential to achieve bacterial clearance without causing excessive tissue damage during the control of the infection.

Macrophages are the first cells to encounter *P. aeruginosa* in the lung [62]. They are important drivers of inflammation during bacterial infection, acting as effector cells and regulators of neutrophil recruitment and life span [82]. Alveolar macrophages are not only responsible for the internalisation and killing of *P. aeruginosa*, but also for the phagocytosis of dying neutrophils, thus limiting neutrophil-induced tissue damage [83]. There is increasing evidence to suggest that *P. aeruginosa* is found in the intracellular environment of various types of mammalian cells, including macrophages. Recently, Garai et al. demonstrated that *P. aeruginosa* can initially reside in phagosomal vacuoles and then be detected in the cytoplasm of macrophages, indicating a phagosomal escape. Intracellular bacteria may eventually induce macrophage lysis, in an ExoS-dependent manner. Therefore, T3SS and ExoS, whose expression is modulated by MgtC and OprF, are key actors in the intramacrophage lifestyle of *P. aeruginosa* [84].

The complement system is also necessary for the survival of mice after pulmonary infection with *P. aeruginosa* [49]. The OprF porin in the OM of *P. aeruginosa* acts as a binding acceptor molecule for C3b to initiate the formation of the membrane attack complex (MAC). Mishra et al. demonstrated that C3b binding was significantly reduced in an OprF-deficient *P. aeruginosa* strain [85]. The innate immune system is essential for the control of *P. aeruginosa* infections; however, the relative importance of these pathways and how they are integrated in vivo remain unclear.

### 3.3. The Adaptive Response

The resolution of the acute inflammatory response requires attenuation of pro-inflammatory pathways. Regulatory T cells (Tregs) inhibit the secretion of pro-inflammatory cytokines and secrete anti-inflammatory cytokines, while dendritic cells initiate adaptive responses. If the *P. aeruginosa* infection is not eradicated during the acute phase, it evolves into a chronic infection characterised by a mucoid biofilm [86]. Persistent neutrophil inflammation is accompanied by an increased effector T cells response, with concomitant elevated expression of IFN-γ, IL-6, IL-1β and IL-17 and a decrease in IL-10 and Tregs [49]. The adaptive response to *P. aeruginosa* infection is characterised by a Th2-skewed response with the upregulation of IL-5 and IL-13; higher B-cell sensitivity to IL-4; low levels of IFN-γ and elevated levels of IL-10, which further downregulate IFN-γ, and decrease co-stimulatory molecules in macrophages. This response hinders antigen presentation and the successful immune response of the host against *P. aeruginosa* infections [86]. The release of IFN-γ can improve lung function due to a Th1-like response [82,87,88]. Consequently, IFN-γ induction of alveolar macrophages may mediate the removal of apoptotic neutrophils, preventing further inflammation due to progression into necrosis [86].

A recent review outlined several studies showing that CF patients chronically colonised with *P. aeruginosa* had greater levels of IL-3, IL-4, and secreted immunoglobulin A (Th2 markers) and lower IFN-γ secretion, compared with intermittently colonised patients or those without *P. aeruginosa* [89]. Hence, the enhancement of the Th1 response may reduce inflammation in the lung by decreasing recruitment of neutrophils due to the reduction in the neutrophil chemoattractant IL-8. At the same time, a diminished Th2 response may reduce the formation of immune complexes, helping to decrease tissue damage. In addition, low IL-13 levels may lead to a decrease in mucus production [90]. However, the appropriate balance of the Th1/Th2 response has not yet been elucidated. In recent years, studies have focused on the Th17 response and its role in the mucosal immune response to respiratory pathogens [91]. Several studies in murine models of acute pneumonia have demonstrated the protective effects of the Th17 response against *P. aeruginosa*, reflected in the reduction in bacterial counts within the lungs of mice or superior survival rates of *Pseudomonas* infected mice relative to the control groups (Section 5.2) [92,93,94,95]. In addition, memory Th17 cells can mount a mucosal immune response independent of serotype-specific antibody, as observed in murine models of *K. pneumoniae* infections [96]. These data are important in the context of *Pseudomonal* vaccines, but it is also worth highlighting that Th17 immune responses may only be slightly effective in the absence of the Th1 pathway [97].

Despite promising results on the protective effects of Th17 responses against *P. aeruginosa* in murine models of acute infection, studies have failed to clarify its role in chronic *P. aeruginosa* infections. Bayes et al. highlighted the key role of IL-17 in mouse survival and prevention of *P. aeruginosa* chronic infection [98]. The authors compared the IL-17 responses to two clinical *P. aeruginosa* CF isolates between WT animals and mice lacking the IL-17RA receptor chain using an agar-based model of infection. IL-17ra^−/−^ mice showed a higher rate of infection and greater mortality than the WT mice [98,99]. However, the Th2–Th17 axis in CF may predispose for the development of *P. aeruginosa* lung infection [86]. IL-23 may be an important upstream regulator of IL-17 and the suppression of IL-23 in mice reduced airway inflammation in response to acute or chronic *P. aeruginosa* infection [99]. Hence, it is possible that vaccine-induced Th17 responses may be ineffective in the CF lung and may even exacerbate the neutrophilic airway inflammation of CF [92]. This needs further investigation.

High antibody production followed by immune complex (IC) formation is also a hallmark in *P. aeruginosa* infections. Production of IgG antibodies during chronic *Pseudomonas* infection, especially in CF patients, has been associated with the high expression of NF-κB; however, the response against specific antigens appears to depend on the infection stage, with some antigens provoking a more intense response in the acute phase, while others are more targeted during the chronic stage. For example, the level of specific antibodies increases in the presence of the *P. aeruginosa* mucoid phenotype, and it is associated with poor prognosis [86].

Regardless of *P. aeruginosa* infection, the airway of paediatric CF patients is associated with an elevated level of B lymphocyte differentiation factor (BAFF), indicating that BAFF expression is not specific to *P. aeruginosa* infection and may be a characteristic of the CF airway. Furthermore, despite the presence of this potent B-cell activator, chronic colonisation is common, suggesting that this response is ineffective [100]. The reasons for the inefficient antibody response against *P. aeruginosa* infections remain unclear, and better knowledge of the underlying mechanisms, such as maturation of avidity/affinity, class change, memory formation, and cytokine synthesis, is needed to understand this phenomenon [86].

Pan et al. demonstrated that IL17-γδ T cells are involved in CD19^+^ B cell activation and the production of immunoglobulins during acute pulmonary *P. aeruginosa* infection [101]. Thus, IL17-γδ T cells may help the bacterial clearance and improve survival via innate and humoral immunity. However, Bayes et al. suggested that pathogenesis was unaffected in mice lacking B cells [98], supporting the idea that an elevated humoral response during chronic *P. aeruginosa* infection is not associated with clinical improvement [102]. Indeed, it is suggested that the high expression of specific anti-*P. aeruginosa* IgG may lead to the formation of circulating immune complexes, which are deposited in the lower airways tissue, triggering tissue damage and long-term deterioration of lung function [102].

Immunoglobulin A (IgA) is also of great importance in the humoral response against *P. aeruginosa* respiratory infections, as it is the predominant antibody isotype in the mucosal immune system, lining in the respiratory tract [103]. The concentration of secretory IgA against *P. aeruginosa* in nasal secretions and saliva correlates with the infection status of CF patients (i.e., not colonised, intermittently colonised or chronically infected with *P. aeruginosa*) [104,105].

The role of B cells in the defence against *P. aeruginosa* is still far from being understood. However, high antibody production is a characteristic of *P. aeruginosa* infections, which has not only proven to be ineffective in the clearance of the pathogen but also to be detrimental when the infection becomes chronic. A better understanding of the humoral response, such as IgG avidity for antigens or the role of IgA in mucosal immunity, is needed to find better methods of diagnosis and treatment of acute and chronic infections generated by *P. aeruginosa,* thus reducing the morbidity and mortality of susceptible individuals such as CF patients.

### 3.4. The Importance of Novel Animal Models

The study of the host immune response to chronic *P. aeruginosa* infection has been difficult in the absence of mouse models capable of developing spontaneous lung disease. CF mouse models do not develop distinctive features such as mucus plugging, chronic bacterial infections, or persistent inflammation [99,106], which led to the generation of larger animal models such as pigs and ferrets [106]. New-born CFTR-KO pigs and ferrets do not have severe lung disease; however, within weeks or months after birth, they spontaneously acquire bacterial infections in the lung [107]. The disadvantage of these models is the difficulty of keeping them alive long enough [108]. Some mouse strains, such as congenic mouse models with the C57BL/6 background, can survive longer to intestinal disease and are susceptible to lung infection with pathogens, including the more persistent *P. aeruginosa* [108]. Cigana et al. refined a murine model of chronic pneumonia extending *P. aeruginosa* infection to three months. The authors used CFTR-deficient mice, which they chronically colonised using *P. aeruginosa* embedded in agar beads by intratracheal instillation. The persistence of *P. aeruginosa* had a greater effect on inflammation and lung damage than the *Cftr* mutation itself, deepening our understanding of the pathogenesis and progression of CF airway disease and *P. aeruginosa* chronic infections [109]. Embedding *P. aeruginosa* in agarose or agar beads physically retains the bacteria in the airways and creates an environment that mimics the biofilm of bacteria and microaerobiosis present in the CF lung. Such retention prevents physical elimination and leads to the persistent stimulation of host defences typical of CF [110]. Although several mouse strains and larger animal models have been used to study chronic *P. aeruginosa* infections, there is no single animal model that fully reproduces the complexity of CF in humans and therefore provides the real picture of the development of *P. aeruginosa* infection in the patient [108].

## 4. Vaccination against *P. aeruginosa*: An Overview of the Last 50 Years

**Figure 3:**
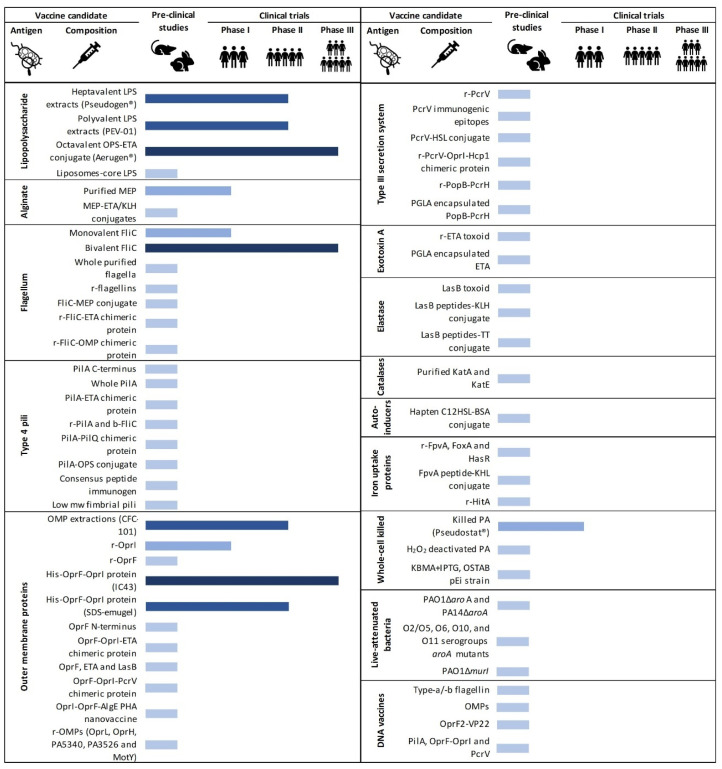
Progress in the development of a vaccine against *P. aeruginosa* infections. The darker the colour of bar, the further the vaccine candidate was evaluated.

Scientists have been pursuing an effective vaccine against *P. aeruginosa* for over half a century, and several antigens and strategies have been examined. However, despite extensive efforts, there are no approved *P. aeruginosa* vaccines to date. The vast majority of vaccines have been evaluated in preclinical studies (Table 1), and while some have progressed to phase I and II trials, only three vaccine candidates have reached phase III trials (Figure 3), namely His-tagged outer membrane protein hybrid OprF-OprI protein (IC43), a bivalent flagellin preparation and an octavalent *O*-polysaccharide-exotoxin A conjugate (Aerugen^®^) (Table 2). There are a range of factors that may have hindered the development of an effective *Pseudomonas* vaccine. The complexity of its pathogenesis, the diverse function of its virulence factors, its high degree of plasticity within the lung and the high diversity of serotypes are considerable obstacles. Furthermore, the respiratory tract of CF patients is particularly complex [111].

### 4.1. Lipopolysaccharide

Lipopolysaccharide is possibly the most widely characterised and investigated vaccine candidate due to its surface accessibility and high immunogenicity. The first LPS vaccine created was a heptavalent formulation based on LPS extracts from seven different strains (Pseudogen^®^, Parke-Davis, Detroit, MI, USA) [222], which was tested in burn patients and in people with cancer or CF [198,199,223,224]. Unfortunately, although patients’ LPS-specific serum antibody titres significantly increased and were sustained for six months, the vaccine showed unacceptable levels of toxicity, and neither eradication of *P. aeruginosa* nor clinical benefit was achieved [198,199]. Similar disappointing results regarding effectiveness and toxicity were obtained with the PEV-01 vaccine (Burroughs-Wellcome, London, UK), which consisted of extracts from the broth culture of 16 strains of different LPS serogroups [112,200,201,225]. Nonetheless, these studies established the potential of LPS as a vaccine antigen candidate and led to the development of new approaches that overcame toxicity. Introduction of complete-core LPS in liposomes induced strong antibody responses while being non-toxic and non-pyrogenic [116,226], but use of detoxified forms of LPS has been most investigated since the 1980s. A lipid A-free LPS vaccine, consisting of large molecular weight fractions of the *O*-antigen, elicited protective antibodies on its own and was non-toxic, non-pyrogenic and immunogenic in pre-clinical studies [227,228]. In contrast, another lipid-A free vaccine was less immunogenic and less protective than untreated LPS when tested in guinea pigs [118]. Nevertheless, several heterogeneous OPS-based *P. aeruginosa* serotypes are responsible for life-threatening infections, limiting the utility of monovalent vaccines. Consequently, a heptavalent version with different OPS serotypes was prepared; however, antagonistic reactions resulted in limited levels of opsonic antibodies being detected [229].

A conjugate vaccine formed by the OPS from *P. aeruginosa* immunotype 5 conjugated to ETA was safe upon intraperitoneal administration to mice or guinea pigs, and was not subject to toxic reversion when exposed to physiologic temperatures [113]. When tested in 20 healthy volunteers in a phase I trial, it was safe and produced anti-ETA IgG neutralising antibodies and anti-LPS IgG opsonic antibodies [230]. A heptavalent formulation of OPS-ETA vaccine was also tested in a phase I study and showed similar promising results [202]. Based upon these findings, an octavalent OPS-ETA conjugate vaccine, called Aerugen^®^ (Crucell, Leiden, the Netherlands), containing the most frequently encountered serotypes in the clinical setting, underwent phase II evaluation with 59 healthy volunteers [203]. The single intramuscular dose was well tolerated and significantly elevated both anti-ETA IgG antibody and anti-LPS antibodys levels to all eight serotypes, but the magnitude of the immune response among the various serotypes was quite variable [203]. In a follow-up study, the ability of this conjugate vaccine to protect 30 young patients with CF was evaluated, and although a single dose was sufficient to elicit antibodies to all vaccine constituents, a subsequent drop-off in antibodies was detected [231,232]. After 10 years of monitoring, it was concluded that (i) affinity rather than quantity of IgG antibodies was the principal factor in the vaccine’s protective capacity [114,233,234]; (ii) yearly boosters were needed to maintain good levels of protective antibodies [204]; (iii) reduction in the frequency of *P. aeruginosa* chronic infection (35% vs. 72% in immunised vs. non-immunised patients, respectively) was associated with better preservation of lung function [114,115,204]; and (iv) cell-mediated immunity was also important for protection [115]. Despite the encouraging results, it failed to show enough efficacy in a randomised, placebo-controlled phase III study involving 476 CF patients, since there was no difference between the vaccinated and control groups, leading to the cancellation of its further development [205].

Conjugates of OPS to different carrier proteins, such as bovine serum albumin (BSA) [235], tetanus toxoid (TT) [236,237] or diphtheria toxoid [238], were investigated. These conjugates enhanced antibody production when compared to OPS or carrier protein alone in pre-clinical studies; however, they were not tested in clinical trials and were developed with only one serotype, limiting the range of protection.

### 4.2. Alginate

The immunogenicity of alginate, also called mucoid exopolysaccharide (MEP), was not realised until the discovery of naturally elicited anti-MEP antibodies in CF patients in the 1980s [239,240,241], which rationalised the development of vaccines targeting alginate. Moreover, despite MEP having strain-specific epitopes, cross-reactivity between strains associated with conserved epitopes was detected [239,240,242], making it an attractive vaccine candidate. Further studies immunising mice, rabbits, rats and guinea pigs with purified MEP extracts also demonstrated the immunogenicity of alginate and the opsonophagocytic killing activity of the anti-MEP antibodies [117,118,119,240,243]. This opsonophagocytic activity was also detected in older non-colonised CF patients [244].

Both molecular size and dose of alginate are key parameters for eliciting opsonic killing antibodies after immunisation with this exopolysaccharide [245]. A trial with healthy volunteers showed that only a vaccine composed of high molecular polymers of MEP raised IgG titres, which were maintained up for two years [206]. Although safe and well tolerated, the immunogenicity of MEP in humans was moderate, with only 35% of the volunteers producing measurable titres [206]. This unsatisfactory outcome led researchers to develop strategies that enhanced immunogenic properties of alginate, such as conjugation to several proteins that would transform it into a T-cell-dependent antigen. A depolymerised and de-*O*-acetylated form of alginate was covalently linked to ETA [120]. It proved to be non-toxic, non-pyrogenic, and evoked significantly better anti-alginate antibody responses than untreated alginate in both mice and rabbits, although effective protection could not be demonstrated [120]. It was later shown that *O*-acetyl groups contribute to the binding epitope of MEP-specific opsonic antibodies [246]. Subsequently, to avoid loss of binding epitopes, the native, non-depolymerised and non-deacetylated alginate linked to keyhole limpet hemocyanin (KLH) was evaluated, which induced opsonic IgG antibodies broadly reactive to heterologous mucoid strains [121]. A polymannuronic acid-type-a flagellin conjugate vaccine constructed by Campodónico et al. enhanced the production of specific IgG antibodies to both antigens and protected mice against mucoid and non-mucoid strains, without interfering with TLR5-mediated immunity [129]. Later, native high-molecular weight alginate was conjugated to type-b flagellin, which elicited high MEP-specific IgG titres with opsonophagocytic killing activity, but low titres against flagellin [130]. Similar encouraging results were obtained when different forms of MEP were coupled to TT [247], a synthetic peptide that contained both B- and T-cell epitopes based on prediction models [248], diphtheria toxoid [249], OMVs of *Neisseria meningitidis* serogroup B [250] or PLGA (polymer poly-lactic-co-glycolic acid) nanoparticles [251].

### 4.3. Flagellar Antigens

The flagellum is a key virulence factor for the successful establishment of *P. aeruginosa* infection and it showed high protection in pre-clinical studies with a mouse burn model [122,123]. Consequently, flagellar proteins, and more extensively, the major component flagellin (FliC), have long been considered as potential vaccine candidates against *P. aeruginosa* infections. In 1991, a phase I clinical trial on 220 healthy volunteers vaccinated intramuscularly with six different monovalent flagellar preparations showed no adverse side effects, and induced high and long-lasting antibody titres in both serum and secretory immune system (SIS) in all individuals [207]. In 1995, Döring et al. vaccinated 10 subjects with three intramuscular injections of a monovalent type-b flagellin vaccine, in an open phase II study and detected long-lasting IgG, IgA, and secretory IgA anti-flagella antibodies [208]. Finally, in 2007, a randomised, double-blind, placebo-controlled, multicentre phase III clinical trial with 483 European CF patients was carried out on a bivalent vaccine containing three type-a subtypes and type-b serotype which reported high and long-lasting serum anti-flagella IgG titres [209]. *P. aeruginosa* strains exhibiting flagella subtypes included in the vaccine were significantly less frequently isolated from vaccinated volunteers than from placebo controls [209]. However, since other flagella types not related to the vaccine antigens were detected, it is clear that this bivalent vaccine was not optimal. Consequently, the degree of protection against *P. aeruginosa* acute infection was determined to be 34% of participants, while 51% of patients with a chronic infection were protected. Ultimately, the primary outcome of 66% protection was not achieved [209].

Since then, few advances have been made with flagella alone as a vaccine antigen. Intact polymeric flagella, which also contains FliD cap protein and other basal body compounds, was superior to monomeric FliC at inducing protective antibodies in a mouse *P. aeruginosa* pneumonia model, achieving 83.3% survival after 125 h [124]. Nevertheless, the use of flagella from PAK (type a) and PAO1 (type b) strains as vaccines did not provide sufficient protection against clinical isolates, and therefore, flagellar antigens are likely to require additional components beyond proteins from prototype strains [124]. Two studies concluded that the N-terminal domain of flagellin was immunogenic, and the antibodies raised were able to recognise the whole bacteria but not heterologous strains [252,253]. Recombinant type-a FliC [126], type-b FliC [127], or a combination of both [128], showed an ability to protect a murine burn model against *P. aeruginosa* challenge, generating robust immune responses. For instance, a recombinant type-a FliC induced both cellular (IL-4 and IFN-γ) and humoral (IgG and IgM) immune responses in mice [125].

Combinations of flagella with other immunogenic compounds of *P. aeruginosa* may be a better approach, and various vaccines have been tested in animal models, such as (i) type-a and -b flagellins with entire polymannuronic acid alone or with whole alginate [129,130], (ii) flagellin (N-terminal domain) conjugated to exotoxin A (domains I and II) [131] and (iii) OprF and OprI outer membrane proteins with type-b flagellin alone or both flagellins [132,133,134]. They produced protective antibodies; however, although survival rates were often high when animals were challenged with the laboratory strains used to obtain the antigens, these rates decreased significantly after challenge with clinical strains.

### 4.4. Type 4 Pili

Because of its accessibility and its role in the early steps of infection, T4P is an attractive vaccine target, and several major pilin (PilA)-based approaches have been evaluated. Ideally, a pilin-based vaccine should target conserved regions, such as the N-terminal domain, to ensure broad protection, but the hydrophobicity and poor accessibility of this domain prevents it from being a good vaccine candidate [254]. Because the C-terminal receptor binding site is structurally conserved, irrespective of variations in the amino acid sequence, it was expected to be a suitable antigen; however, pre-clinical studies showed inconsistent results in achieving cross-reactivity and significant protection [135,255]. This may be because (i) display of functional binding sites is limited to the tip of the pilus [256]; (ii) the receptor binding domain is not surface exposed [257,258,259], and (iii) T4P is subdivided into five distinct serotypes, with most clinical isolates possessing pilins from group I [260]. Nevertheless, antigenicity of this region can be improved by conjugation to carriers. Two chimeric proteins were constructed through insertion of different sizes of the DSL region of PAK strain in a non-toxic form of the ETA [138,139]. When administered subcutaneously into rabbits, it produced antibodies against both antigens, which reduced bacterial adherence and neutralised the cell-killing activity of ETA [138]. Intranasal immunisation of rabbits with the chimeric protein generated both pilin specific serum IgG and salivary IgA immune responses, but the authors suggested that a cocktail of pilin DSL sequence chimeras might achieve a broader effective vaccine [139].

Intact PilA has also been investigated, either alone or in combination with other antigens. Mice intratracheally immunised with pilin protein showed a significant improvement in survival (80–100%) in response to challenge, and the bacterial load was reduced up to 5 logs after 18 h [136]. However, survival rates were lower (23.5–47%) when mice were challenged with clinical strains, compared to the parent strain (PAO1). Interestingly, subcutaneous immunisation showed very low rates of survival (20%) [136]. When recombinant PilA was administered to mice with alum and naloxone as adjuvants, it elicited effective cellular and humoral immune responses, including increased IL-17 secretion and antibody-mediated opsonophagocytic killing. Survival rates of 75% were observed when mice were challenged with PAO1 and a clinical isolate [137].

The *P. aeruginosa* strain 1244 naturally presents an *O*-antigen repeating unit covalently linked to each pilin monomer. When mice were immunised with a strain 1244 PilA-glycoprotein-based vaccine, it provided *O*-antigen-specific protection via the mucosal and systemic routes of immunity [144]. In a burn wound model, a bivalent vaccine composed of type-b FliC and PilA elicited robust cellular and humoral responses (consisting of a Th2 response with high levels of subtype IgG1 and lower levels of IgG2a and cytokine response of IL-4, INF and IL-17), and proved quite protective, with a 3 to 4 log reduction in bacterial load in the sites of infection within 24 h of challenge [140]. Moreover, immunised mice showed 87% survival against challenge with a clinical isolate from a burn patient [140]. More recently, this group developed a trivalent vaccine by incorporating a type-a flagellin to the previous bivalent vaccine. This was superior in terms of survival rates (50–83% vs. 92–100%) and activation of opsonophagocytic activity (43–88% vs. 89–93%) [141]. These results should encourage researchers to test such vaccine approaches in animal models of respiratory infections.

Synthesis of consensus peptide immunogens that represent the various pilin serotypes to achieve broad protection without the use of a vaccine cocktail has yielded promising results. Cross-reactive polyclonal sera were elicited, generating protective high antibody titres [145,146,261,262,263]; however, further studies to assess its effectiveness against various clinical strains should be carried out. Proteins of the pilin subcomplexes, such as PilQ, have been investigated. Gholami et al. synthesised a recombinant fusion PilQ/DSL-PilA protein that produced high titres of anti-pilin antibodies with opsonophagocytic properties in mice [142,143]. Faezi et al. obtained a recombinant protein using the secretin domain of the PilQ protein (PilQ_380-706_) and a fusion PilQ_380-706_-PilA protein, which produced specific opsonic IgG antibodies in rabbits that inhibited twitching motility, suggesting that they could be used as vaccine antigens [264,265]. Finally, an engineered fimbrial low-molecular-weight protein pili (Flp) that contained dominant immunogenic features induced both humoral and cellular immune responses in mice [147]. However, these studies used prototype strains (PAO1, 6266E) and some did not determine the response to challenge and consequently, additional in vivo and in vitro studies are needed to assess their protective potential.

### 4.5. Outer Membrane Proteins

#### 4.5.1. OprF Major Porin and OprI Lipoprotein

The most widely investigated outer membrane proteins as vaccine antigens are the major porin protein F (OprF) and the lipoprotein I (OprI). Various studies demonstrated that these OMPs are surface-exposed, highly conserved among all different serotype strains and immunogenic [266,267,268], prompting their evaluation as vaccine candidates. Immunisation of mice and rats with highly purified preparations of either OprF or OprI elicited cross-reactive, opsonising and protective antibodies [269,270,271,272,273], but LPS contamination was an issue, making them unsuitable for use in humans. Another vaccine comprising OMP fractions from four *P. aeruginosa* strains (CFC-101) was tested in three clinical trials, showing toxicity issues without clinical benefit [210,211,212]. Recombinant production of these proteins overcame toxicity problems while maintaining vaccine efficacy in pre-clinical models [148,149,150,274], but the degree of protection was lower when compared to LPS-based vaccines [148,270,271]. Immunisation with recombinant OprI antigen in a small phase I clinical trial with 28 healthy volunteers showed considerable variations in the individual antibody responses [213]. Later, a modified His-tagged hybrid protein that contained protective epitopes (Met–Ala–(His)_6_OprF_190–342_–OprI_21–83_) was produced, and resulted in a synergistically enhanced protection in an immunosuppressed mouse model [151]. Subsequently, two different formulations for these vaccines, systemic and mucosal, were investigated [275].

Thirty-two volunteers were vaccinated intramuscularly with the systemic formulation of the His-tagged hybrid protein, which was well-tolerated. Moreover, a significant rise in complement-promoting, opsonising antibody titres against both OprF and OprI was detectable up to six months after the third vaccination [214]. Robust responses were also observed in burn patients, although they required two immunisations and the antibody titres achieved were about 85% that of the healthy volunteers [215]. This OprF-OprI hybrid antigen was initially developed by Intercell AG (Vienna, Austria) and named IC43 (recently renamed as VLA43). In a phase I, randomised, placebo-controlled, blinded trial with healthy adult volunteers, it was concluded that a two-vaccination regimen in a seven-day interval was required for the induction of a significant IgG response [216]. Similar results were obtained in a phase II study in mechanically ventilated ICU patients [217]. Despite the significantly lower rate of mortality in vaccinated patients (35.6–37.6% vs. 42.9%), there was no correlation between the observed mortality benefit and confirmed *P. aeruginosa* infections. Moreover, although the differences were not statistically significant, a higher infection rate was observed in the treatment group (11.2–14%) than in the placebo group (6.1%), suggesting that IC43 vaccine may affect *P. aeruginosa* virulence rather than clearance [217]. A confirmatory, randomised, multicentre, placebo-controlled, double-blind phase II-III with 799 ICU patients, showed that although IC43 was both well-tolerated and immunogenic, there was no clinical benefit over placebo group in terms of mortality [9]. Several factors may have led to this failure: (i) the intrinsic heterogeneity of ICU patients, (ii) additional variability due to the introduction of patients from different European countries, and (iii) *P. aeruginosa* infection prior to the development of effective IgG immune response [217]. Recently, a heptamer of IC43 was evaluated, which resulted in increased levels of antigen-specific IgG, reduced lung damage and higher survival rates than IC43 alone (80% vs. 30%). It was also shown to be moderately protective within 12 h, with one log reduction in bacterial burden. These improvements may be due to the exposure of a higher number of immunogenic epitopes [152].

The mucosal formulation of the OprF-OprI hybrid protein vaccine was examined as an intranasal vaccine in eight healthy volunteers, and showed elevated IgG and IgA levels in the airways, which persisted for up to one year and had no side effects [218,219]. A systemic booster following a primary mucosal vaccination enhanced the moderate immune response obtained through single nasal application by raising levels of systemic IgG antibodies [219,276]. Similarly, when this nasal-systemic immunisation schedule was performed in patients with pulmonary disorders, elevated IgG2 and IgG1 antibody titres in serum and saliva were observed. However, although high antibody titres persisted for six months post-vaccination in the airway mucosa, serum levels notably dropped in all patients [277]. Mannose-modified chitosan microspheres loaded with this vaccine showed promise as a delivery system, since nasal immunisation of mice demonstrated 75% protective efficacy and a strong specific humoral response, including IgA titres in nasal washes, bronchoalveolar lavage (BAL), and intestinal lavage and systemic IgA and IgG titres in serum [153].

Other approaches involving OprF and OprI include mice immunisation with the N-terminal domain of OprF, which elicited specific and protective IgG1 and IgG2 antibodies [154]. Co-administration of OMPs with other antigens was performed as early as 1993, with administration of combinations of OprF, elastase, and ETA toxoid in a rat model of chronic pulmonary infection, although combinations with elastase or ETA toxoid did not improve the protective efficacy provided by OprF alone [156]. A chimeric OprF-OprI-ETA antigen enhanced macrophage phagocytosis of various *P. aeruginosa* strains, produced antibodies that neutralised ETA cytotoxicity, and conferred protection in a burn wound mice model (60–80% survival after 10 days) [155]. Both OprF and OprI combined with either type-a or type-b flagellins elicited protective IgG antibodies in mice and monkeys [132,133,134]. Finally, OprF and OprI combined with PcrV (a T3SS protein, Section 4.6) administered to a burn mouse model were highly protective, showing up to seven log reduction in bacterial burden within 12 h after challenge and survival rates of 75% [157].

Viral vector systems presenting protective epitopes of OprF have provided encouraging results with strong IgG and/or IgA antibodies titres and protection against *P. aeruginosa* challenge demonstrated in mice immunised with chimeric viruses expressing different epitopes. Influenza virus [278], cowpea mosaic virus [279,280], tobacco mosaic virus [281] and adenovirus [282,283,284,285] were used as vectors. Finally, a polyhydroxyalkanoate (PHA) nano-vaccine with *P. aeruginosa* cellular inclusions engineered to display OprF, OprI and AlgE antigens in the surface induced a protective Th1-cellular response associated with the production of antibodies that reacted with different strains and possessed opsonophagocytic killing activity [158].

#### 4.5.2. OprL, OprH, OprG and Others

Other immunogenic OMPs have been evaluated more recently. OprL was identified as an early immunogenic protein in CF patients [286] and elicited strong IL-17 secretion in a murine pneumonia model [92]. Vaccination with either the whole recombinant OprL or a recombinant fragment conferred Th17-dependent and serotype-independent protection in mice [159,160]. A screen of two LPS-heterologous *P. aeruginosa* strains in a murine acute pneumonia model showed that porin protein OprH was the most highly expressed OMP, but immunisation of mice with OprH and curdlan adjuvant (Section 5.2) rendered no significant protective efficacy, nor did co-administration of iron acquisition proteins or a prepared trivalent mixture [161]. In contrast, vaccination with OprH refolded in DHPC micelles elicited specific opsonic antibodies and conferred protection against the two strains used [161], highlighting the importance of the final vaccine formulation in eliciting the response. In a limited, exploratory study, high OprH-specific IgG and IgM antibodies levels were found in chronically infected CF children. Additionally, although OprH showed the lowest IgA response in BAL, it was still higher in CF children without a history of positive *P. aeruginosa* culture [287]. In contrast, a considerable response to OprG was only detected in serum IgA levels in children with chronic lower respiratory tract infection [287]. Mice vaccinated with a recombinant OprH showed 46.15% survival against *P. aeruginosa* infection [288]. Recently, Bianconi et al. identified various outer membrane and periplasmic proteins, with both known and unknown functions, that significantly increase survival rate among challenged mice when given in combination. Three of these, OMPs MotY, PA5340 and PA3526, gave the maximum protection (50% survival) [6].

### 4.6. Type III Secretion System

PcrV was the first protein of the T3SS translocation apparatus (PcrV) used as a vaccine antigen, which resulted in protective immunity and enhanced survival in challenged mice, both in lung infection and burn models [162,289]. Immunisation of immunocompromised mice with full-length PcrV or five differently truncated PcrV proteins showed that the most immunogenic regions were the Mab166 epitope (PcrV_144-257_) and the carboxyl terminus. Indeed, immunisation with antigens containing these regions provided comparable survival rates with immunisation with whole PcrV (65%), with a significant increase in IgG1 and IgG2 titres [165]. Comparison of recombinant PcrV administered with different adjuvants (alum and CpG) showed an increase in IgG1 and IgG2a titres, indicative of a Th1/Th2 biased response [93]. Intranasal administration of PcrV-alum and PcrV-CpG vaccines raised IgG and IgA titres in serum and BAL of mice. The survival rate of PcrV-CpG immunised mice was 75%, compared to 29% for PcrV-alum [163]. An optimised soluble PcrV derivative (PcrV_NH_) combining two immunodominant domains of PcrV triggered a Th2-mediated immune response, and while no mice in the unimmunised group survived, 50–60% of immunised mice were protected [166]. However, the survival rates were still low, probably because it did not efficiently elicit Th1 and Th17 responses, but this limitation could be addressed by adjuvant optimisation or combination with other antigens [166]. Overall, variable survival rates for PcrV alone have been reported, ranging from 20% to 75% [157,163,164,168].

A recombinant PcrV with a L-homoserine lactone autoinducer showed over 75% survival when tested in a burn wound sepsis mice model [167]. A trivalent combination of PcrV with the outer membrane lipoprotein OprI and the T6SS effector Hcp1 administered with Al(OH)_3_ triggered a Th2-biased immune response and enhanced protection over immunisation with a single unit of the three antigens in an acute pneumonia mice model (90% vs. 40–50% survival) [168]. Finally, as previously mentioned (Section 4.5.1) the chimeric OprI-OprF-PcrV chimeric vaccine elicited sustained IgG titres for 235 days after the second booster in a burn mice model and improved the survival rate of challenged mice over those that were immunised with individual antigen showing up to seven log reduction in bacterial challenge within 12 h [157].

Other proteins of the T3SS translocation apparatus have also been evaluated. A reverse vaccinology approach identified that PopB possesses strong T-cell epitopes and non-protective B-cell epitopes [92]. Intranasal immunisation of mice with PopB mixed with the Th17 adjuvant curdlan induced a systemic and mucosal Th17 response and was partially protective, despite no opsonic antibodies being induced [92]. Comparable Th17 responses and mice protection were obtained with recombinant PopB with the chaperone protein PcrH encapsulated in PLGA nanoparticles [94].

### 4.7. Extracellular Products

#### 4.7.1. Exotoxin A

Widespread expression of this exotoxin by most clinical *P. aeruginosa* isolates [290,291] and detection of anti-ETA antibodies in patients with bacteraemia experiencing better outcomes [292,293] rationalised the use of immunogenic and non-toxic forms of ETA in vaccine development. Several attempts to obtain a detoxified version have reported issues around instability and toxicity after storage and incomplete inactivation or unacceptably low levels of immunogenicity in mice and rats [294,295,296,297,298,299,300]. A semi-purified attenuated ETA showed protection in a mouse burn model; however, it contained trace amounts of LPS and OMPs and an ETA-specific response was not demonstrated [300]. Different recombinant exotoxins without ribosylation activity elicited acceptable levels of antibodies and conferred protection in animal burn models [169,170,171]. More recently, an ETA-PLGA nano-vaccine candidate showed enhanced production of cytokines and IgG antibodies when compared to vaccination with ETA alone [172]. Mixed results were obtained overall using a single-component ETA-based vaccine, and hence the effectiveness of such a vaccine is questionable. Nevertheless, ETA has been used as carrier protein for other antigens, such as LPS, alginate, OMPs and flagella, showing improved immunogenicity [120,131,156,202].

#### 4.7.2. Other Extracellular Products

An elastase toxoid created by site-directed mutagenesis showed good levels of protection in mice [173], while an elastase peptide-KLH conjugate showed potential to reduce the severity of lung disease in a rat *P. aeruginosa* chronic lung infection model [174]. When such epitope peptides were conjugated to TT, antibody titres were at least double that of the KLH conjugation [174]. Although addition of elastase to OprF did not improve protection in a rat lung model [156], some studies reported that addition of elastase, alkaline protease and ETA toxoids to a vaccine improved its protective efficacy in different animal models [301,302,303]. A vaccine comprising a previously identified common protective antigen (OEP) that contained the protein moiety of the OM endotoxin along with ETA, elastase, and protease increased antibody titres to all four of the antigens in patients with diffuse panbronchiolitis, which were maintained for up to six months after vaccination [304].

### 4.8. Other Bacterial Components

Although surface-exposed and -secreted bacterial components comprise the majority of vaccine antigens evaluated, bacterial proteins that are not usually surface exposed have also shown protection. Despite its usual periplasmic and cytoplasmic location, KatA catalase showed a significantly greater clearance of both homologous and heterologous strains in mucosally immunised rats, which correlated with an increase in anti-KatA IgG and IgA titres in BAL [175]. They later identified four periplasmic proteins (Pa13, amidase, aminopeptidase and KatE catalase) that demonstrated enhanced bacterial clearance in a rat model [176]. KatA, KatE and Pa13 immunisation also led to the recruitment of phagocytic cells in the rodent model [177]. KatA combined with amidase was protective in a chronic lung infection model [10], and, in a limited, exploratory study, high serum IgG and IgM levels towards KatA were observed in chronically infected CF children [287].

Iron uptake system proteins, including FpvA, FoxA, and HasR were highly expressed during infection in a murine acute pneumonia model [161]. Unfortunately, and despite FpvA eliciting a Th17 response in mice [92], they did not show protective efficacy in this model when administered separately or in combination [161]. Although FpvA peptides administered alone were poorly immunogenic, their conjugation to KLH elicited both mucosal (IgA) and systemic (IgG, IgM, IL-17) responses when given intranasally, decreasing bacterial load in an acute murine pneumonia model [179]. Unfortunately, the high genetic variation between CF isolates of the *fpvA* gene [305] may hamper its coverage.

Recently, HitA, a ferric iron-binding periplasmic protein induced high levels of IgG1 and IgG2a specific antibodies in mice when administered subcutaneously in combination with a high dose of BCG plus IFA, followed by two booster doses of combined HitA and IFA [180].

Vaccines involving components of the QS system seem to have limited potential. Immunisation of mice with the autoinducer of the Las QS system (a homoserine lactone, C12HSL) conjugated to KLH [306], KatA [10] or PcrV [167] significantly increased IgG titres and/or reduced bacterial burden, subcutaneous administration in mice a C12HSL hapten conjugated to BSA with Freud’s adjuvant showed only a 36% survival [178].

### 4.9. Whole-Cell Killed and Live-Attenuated P. aeruginosa Vaccines

Construction of a vaccine using whole-cell killed or live-attenuated bacteria is a less developed strategy for *P. aeruginosa*. Although an orally administered live *P. aeruginosa* PAO1 strain failed to protect immunised rats against subsequent pulmonary infection [307], mucosal and systemic immunisation with a paraformaldehyde-killed strain (Pseudostat^®^) induced protective immune responses in the lung, enhanced bacterial clearance (>90%, 4 h after challenge) and reduced mortality [181,182,183]. Oral immunisation of nine bronchiectasis patients with this preparation showed a significant reduction in bacterial burden in the sputum and specific lymphocyte responses [220]. In a phase I study with 30 healthy volunteers, it induced a significant serum IgA response and increased functional opsonisation and killing by macrophages [221]. Nevertheless, neurological, gastrointestinal, and respiratory disorders were detected in 20 individuals [221]. Recently, the use of H_2_O_2_ to deactivate *P. aeruginosa* strains showed potential, resulting in reduced toxicity and retention of more complete epitopes compared to formaldehyde deactivation. Indeed, it stimulated opsonic protection (IgG1 and IgG2a) and induced IgG3 at later stages and IgM at earlier stages in mice [184].

Live-attenuated vaccines have also been developed. An *aroA* deletion mutant of *P. aeruginosa* (PAO1Δ*aroA*), which is deficient in aromatic amino acids synthesis, was safe and enhanced titres of opsonic antibodies, primarily directed against the *O*-antigen [186], and five of the seven protocols tested engendered 100% protection against LPS-homologous strains in an acute lethal pneumonia mice model [187]. By contrast, a live-attenuated vaccine constructed with PA14 strain (PA14Δ*aroA*) protected mice and rabbits against lethal pneumonia caused by LPS-heterologous strains [188]. When PAO1Δ*aroA* was tested in a murine corneal infection model, outer membrane antigens, but not the LPS *O*-antigen, were identified as the immune effector, providing broader coverage against LPS diverse strains [308]. Mucosal vaccination with a multivalent vaccine composed of live-attenuated *P. aeruginosa* strains, including PAO1Δ*aroA,* stimulated a multifactorial response, involving both humoral and cellular elements without provoking immunologic interference [189]. Recently, Cabral et al. used a live auxotrophic strain (PAO1Δ*murI*) lacking an enzyme involved in D-glutamate biosynthesis, an essential component of peptidoglycan. Both humoral and cellular responses were triggered when delivered intranasally in a two-dose vaccination schedule, leading to murine survival of 86–88% against lethal pneumonia [190].

Since live-attenuated vaccines may retain residual virulence that could make them unacceptable for human use [309], Meynet et al. developed a safer approach based on the killed but metabolically active (KBMA) attenuation method [185]. This KBMA *P. aeruginosa* cannot replicate in the host and was designed to overexpress the T3SS. In a murine acute pulmonary infectious model, it was safe and elicited a strong humoral response with high antibody titres to OprF and PcrV, as well as activation of the Th1, Th2, and especially Th17 pathways [185].

### 4.10. DNA Vaccines

DNA vaccines are increasingly being investigated for many vaccine applications as their ease of development and stability offer considerable advantages over protein sub-unit vaccines. DNA vaccines encoding type-a or b flagellins from different serotypes showed strong immunogenicity and efficiently protected mice against challenge with homologous but not heterologous strains in a murine *P. aeruginosa* pneumonia model [191]. Antibodies preventing TLR5 activation were also raised, but a DNA vaccine encoding a mutant *FliC* that reduced its ability to interact with TLR5 while maintaining immunogenic properties overcame this issue. DNA immunisation with OprF also showed potential, eliciting OprF-specific IgG1 opsonic antibodies that reduced bacterial burden in the lungs of a mouse model of chronic pulmonary infection [192]. Protective capacity was substantially enhanced by one of two strategies: (i) a booster immunisation using a chimeric influenza virus that contains an immunoprotective epitope of OprF, or (ii) immunisation with a DNA vaccine that contained an OprI-OprF gene-fusion construct [193,194]. Recently, Gong et al. compared four DNA vaccines with *oprL* and *oprF* genes as monovalent vaccines, a fusion OprL-OprF vaccine or a divalent combination (OprL and OprF). They all induced a humoral response based on serum antibodies, IL-2 and IFN-γ, and protected chickens against *P. aeruginosa* challenge, with the divalent combination showing the greatest survival (80%) [195]. A Herpes simplex virus type 1 (HSV-1) protein (VP22) was used to enhance the immunogenicity of an OprF DNA vaccine against *P. aeruginosa,* which elicited IFN-γ and IgG2a antibodies, but failed to protect mice against *P. aeruginosa* challenge (0–40% survival) [196]. The feasibility of ETA DNA vaccines has been demonstrated by immunisation of mice with a mutant *toxA* gene encoding a non-cytotoxic product, which stimulated production of neutralizing antibodies that protected mice against challenge with the wildtype ETA [310,311]. Later, a novel DNA vaccine that simultaneously contained ETA and PcrV encoding genes confirmed its immunogenicity and ability to protect mice against *P. aeruginosa* challenge [312]. Finally, a plasmid encoding PilA incorporated in a multivalent DNA vaccine that also contained other plasmids encoding a fusion OprF-OprI fusion and PcrV protein showed 100% survival in the mouse model of *P. aeruginosa* pneumonia when administered via electroporation [197]. DNA vaccines are worthy of further consideration due to their potential to induce a wider range of immune responses, safety and stability.

## 5. Adjuvants

### 5.1. Definition, Function, Classification, and Licensing

Vaccine adjuvants enhance and/or shape antigen-specific immune responses. Currently, subunit vaccines are attractive due to their high safety profile; however, they show a reduced immunogenicity which necessitates the inclusion of adjuvants [313,314,315]. Adjuvants have facilitated the development of vaccines against pathogens for which live-attenuated or inactivated vaccines are ineffective. Therefore, correct identification and selection of an appropriate adjuvant is crucial in the development of new effective and safe vaccines [315].

Adjuvants can be classified as delivery systems (DS) and immunostimulators (IMS), or a combination of both. DS work as carriers with which antigens are associated, creating local pro-inflammatory responses that recruit innate immune cells to the injection site. This type of adjuvant is well represented by liposomes or virosomes [316]. Immunostimulators, on the other hand, include innate immune receptor ligands such as TLRs, NLRs, C-type lectins, and retinoic acid I-inducible gene (RIG-I). Among the more advanced compounds are the TLR4 MPL ligand (AS04), which is part of the adjuvant system of the Cervarix HPV vaccine (from GSK), and the CpG oligodeoxynucleotide (ODN) of the TLR9 ligand [313,316].

Although many molecules have adjuvant effects, only a handful have been licensed for use in humans [317]. Aluminium salts (also known as alum) were the only approved adjuvant for more than a century, and they continue to be the most widely used. Recently, other adjuvants have been approved for use in human vaccines such as MPL (a detoxified form of bacterial lipopolysaccharide), oil-in-water emulsions (MF-59), combinations of AS (adjuvant systems), e.g., AS03 is used in pandemic influenza vaccines (Pandemrix, Arepanrix, Adjupanrix, GSK). Finally, virosomes, spherical lipid layers assembled in vitro with viral proteins to resemble viral membranes, are currently used in influenza and hepatitis A human vaccines [317,318]. The approval of a few adjuvants for use in human vaccines has been attributed to the lack of knowledge of their action mechanism. Thus, it is now clear that understanding the mode of action of adjuvants is fundamental for the design of vaccines that produce pathogen-specific long-term memory effectors and to assess the safety of adjuvants at the development and regulatory stages [315].

### 5.2. Adjuvants in Vaccines against P. aeruginosa

A considerable limitation of early vaccine development is that many studies either do not consider the impact of the adjuvant in the *Pseudomonas* vaccine formulation or do not specify the contribution of this molecules to the protective effect of the immunisation. Hence, knowledge about the role of adjuvants in *P. aeruginosa* vaccines is limited, leaving one of the key questions unanswered: what immune response should the adjuvant trigger to provide effective protection against this pathogen? Although this has not been fully elucidated, recent studies have provided great insights (Table 3). In general terms, studies have suggested that mucosal immunity, mostly provided by Th17 immune responses, is essential to achieve protection against *P. aeruginosa* in murine models [92,94], possibly, in conjunction with other pathways such as improved protection by a Th1/Th17 balance [95] (Table 4).

Aluminium hydroxide (Al(OH)_3_) and aluminium phosphate (AlPO_4_), commonly referred to as “alum”, are the most widely used adjuvants in preclinical and clinical trials of *P. aeruginosa* vaccines [137,154,217]. This is not surprising, given that alum was the only adjuvant approved for human vaccines until 1997, when the MF59-adjuvanted seasonal influenza vaccine was first licensed [319,320]. Antigens are adsorbed on alum, improving antigen uptake and presentation by antigen-presenting cells (APCs) [320,321]; furthermore, alum induces inflammasome and recruitment of different immune cells [313]. Al(OH)_3_ was used in the phase II study of the *Pseudomonas* IC43 (OprF/I) vaccine and it did not improve the immunogenicity of IC43 [217]. Seroconversion was highest in the group receiving IC43 without adjuvant (80.6%), while control was only 65.0% in the alum-adjuvanted group [217]. Furthermore, Hamaoka et al. examined the role of alum as an adjuvant for the induction of PcrV-specific immunity in a murine model of intratracheal-induced acute *P. aeruginosa* pneumonia. A marked increase in anti-PcrV IgG1 titres but a decrease in IgG2a titres was observed, indicating a Th2-biased immune response and highlighting a key limitation of alum, it induces strong Th2-mediated immune responses, as a poor inducer of cellular immune responses [93]. As mentioned in Section 3.3, the adaptive response to *P. aeruginosa* infections is characterised by a skewed Th2 response [86], with high levels of Th2 markers (IL-3, IL-4) and low levels of Th1 markers (IFN-γ), as observed in patients with chronic *P. aeruginosa* infections [89]. Thus, adjuvants such as alum that increase the Th2 response may not be optimal for increased protection against *P. aeruginosa*, and may even be detrimental.

Studies in mouse models comparing alum with other adjuvants, such as naloxone (NLX, an opioid receptor antagonist) or deoxygenated lipooligosaccharide (dLOS, a TLR4 agonist derived from an *Escherichia coli* LPS), provide hints about the harmful versus beneficial immune responses against *P. aeruginosa* [137,322]. NLX, in combination with alum and recombinant *P. aeruginosa* PilA (r-PilA), generated robust Th1 and Th2 type responses (IgG1 and IgG2a), compared to the use of rPilA + alum. The addition of NLX did not alter the IL-4 (Th2 marker) production, but it produced a significant increase in INF-γ and IL-17, suggesting that NLX shifts the Th2 response towards a more balanced Th2/Th1 response [137]. The importance of this balanced response was reflected in better protection against *P. aeruginosa* (75% mice survival after 7 days of infection) compared to immunisation with r-PilA + alum (approximately 40%) [137].

The use of dLOS combined with alum (CIA06 system) also improved the immunogenicity of an OMP vaccine. Mice immunised with OMP+ CIA06 showed 60 to 90% survival after 8 days of infection with *P. aeruginosa*, while mice given OMP + alum showed 20 to 50% survival. The improved protective effect of the CIA06 adjuvant may be related to increased opsonophagocytosis, along with elevated Th1 and Th17 cell responses [323].

Another licensed adjuvant evaluated in *P. aeruginosa* vaccines is CpG ODN, a short, single-stranded synthetic DNA fragment containing an unmethylated CpG sequence (CpG motif) that is relatively common in the genomes of most bacteria and DNA viruses [324]. This adjuvant mimics the immunostimulatory effects of bacterial DNA and triggers cells that express TLR9, such as DC and plasmacytoid B cells, which produce inflammatory cytokines and activate NK cells, monocytes, and neutrophils [163]. Intranasal vaccination with PcrV-CpG showed efficacy against *P. aeruginosa* pneumonia, with 73% of vaccinated mice surviving, compared with 30% of the mice vaccinated with PcrV-alum or CpG alone [163]. Anti-PcrV IgG titres (IgG1, IgG2a, and IgG2b) and anti-PcrV IgA titres were significantly higher in mice vaccinated with PcrV-CpG than in the other groups, suggesting a Th1/Th2 response [163]. Previous studies have also demonstrated the efficacy of CpG ODN as an adjuvant in a *P. aeruginosa* vaccine consisting of *O*-polysaccharide serotype IATS-1 coupled to Toxin A [115] and in a DNA vaccine containing the genes encoding ETA and PcrV [312].

Other FDA-approved adjuvants for use in human vaccines that have been used in pre-clinical studies of *P. aeruginosa* infections include AS04 and MF59^®^ [319,325]. However, in a study on a trivalent vaccine, PcrV_28-294_-OprI_25-83_-Hcp_11-162_ (POH), no improvement was observed with either relative with the use of alum in models of murine pneumonia (survival rates and similar antibody titres) [168], consequently they do not stand out as a promising option for more effective *P. aeruginosa* vaccines.

Taken together, it is clear that Th2 cell responses are not optimal to achieve protection against *P. aeruginosa* and that a shift to Th1 or Th17 responses may improve the efficacy of *P. aeruginosa* vaccines. This, together with the importance of mucosal immunity, which is strongly related to the IL-17 family of cytokines on different epithelial barrier surfaces [323], has led to the evaluation of new adjuvants. These adjuvants, including PLGA and curdlan, have shown promising results in terms of their protective effect against respiratory infections caused by *P. aeruginosa*, mainly by increasing Th17 cell responses [92,94].

Curdlan is a component of the inner fungal cell wall, a covalently linked branched glucan moieties β-(1,3) linked to other polysaccharides and proteins. It is a mast cell chemoattractant (MC) [326], mediated by Dectin-1, which activates DC cells directing CD4+ IL-17 differentiation, producing Th17 effector cells [327]. Wu et al. evaluated whether curdlan enhanced the protective effect of the Th17 response-stimulating antigen PopB, against *P. aeruginosa*. Immunisation with PopB/PcrH+curdlan induced a systemic and mucosal Th17 response, but it did not increase the protective effect of immunisation (comparable survival rate, 62.5%). This protective efficacy was antibody-independent but IL-17-dependent [53,92], emphasizing the importance of triggering Th17 responses. Schaefers et al. found that PopB encapsulated PLGA nanoparticles also elicited Th17 responses and that PopB/PcrH with curdlan had a protective effect against *P. aeruginosa* infections. Immunisation with PLGA-entrapped PopB/PcrH or curdlan-PopB/PcrH resulted in Th17 responses three to four times higher than in mice immunised with PLGA or PopB/PcrH alone. This was reflected in the protective effect of immunisation (in contrast to Wu et al.). Six days after infection, mice vaccinated with PLGA-PopB/PcrH or curdlan+PoB/PcrH had significantly better survival (~70% and ~50%, respectively) than mice vaccinated with PopB/PcrH alone (100% mortality) or PLGA alone [94]. These studies highlight that curdlan significantly increases Th17 response, which is critical for the protective effect against *P. aeruginosa*. Schaefers et al. also reported a lack of antibodies with opsonophagocytic killing activity against *P. aeruginosa* suggesting a Th17- mediated protection [92,94].

The mucosal adjuvant LT (R192G/L211A) or dmLT consists of an 84 kDa polymer protein that is distinguished from its parent molecule, thermolabile enterotoxin (LT), by the replacement of two residues in subunit A, a glycine with arginine (R192G) and alanine with leucine (L211A). It induces strong IL-17 secretion and antigen-specific Th17 responses [95,328]. Baker et al. (2019) showed that mice immunised with OMPs+ dmLT produced significantly more antigen-specific IgG and Th1 and Th17 CD4^+^ memory T cells in the lung compared with the control groups (adjuvant alone or sham). Immunisation with OMPs+ dmLT protected the mice against lethal *P. aeruginosa* lung infection, and was associated with the early production of IFN-γ and IL-17 (Th1/Th17 responses). Mice immunised with OMPs +dmLT showed 53% survival in 10 days, while all other groups completely succumbed to infection within 1–3 days. Again, these data highlight the role of Th1/Th17 responses in protection against *P. aeruginosa*. The ability of dmLT to support antibody responses in addition to Th1/Th17 cellular immunity is significant and offers a clear advantage, particularly for those pathogens that require both humoral and cellular responses for protection [95]. Notwithstanding these data, these studies mostly used acute infection models, so the question remains as to whether a Th17 response would be beneficial in chronic infections or if, on the contrary, as stated by other authors [89,92], potentially harmful in these stages of *P. aeruginosa* infections.

The contribution of adjuvants to the protection against chronic *P. aeruginosa* infection has also been examined. Krause et al. evaluated the systemic and mucosal immunogenic properties of a non-human primate-based adenovirus vector, AdC7, expressing *P. aeruginosa* OprF (AdC7OprF) and compared it with a human serotype (Ad5OprF), using an agar-beads chronic infection model in mice [284]. Intramuscular immunisation of mice with AdC7OprF induced similar levels of anti-OprF IgG in serum and mucosa but superior levels of anti-OprF IgA, compared to Ad5OprF vector. AdC7OprF-induced anti-OprF IgG antibodies that were predominantly IgG1 and IgG2b isotypes, followed by IgG2a and IgG3, with lower titres of IgG2a, IgG2b and IgG3 compared to Ad5-immunised animals [284]. Immunisation with either Ad5OprF or AdC7OprF resulted in the protection of 100% of mice against *P. aeruginosa*. The AdC7OprF vaccine induced protective immunity against *P. aeruginosa* despite the presence of lower total systemic anti-OprF IgG titres. Both Ad5OprF and AdC7OprF induced an OprF-specific IFN-γ response in lung CD3^+^ T cells, while IFN-γ levels in mouse-derived CD3^+^ T cells that had received Ad5Null or AdC7Null were low [284]. The direct administration of AdC7OprF to the respiratory tract resulted in increased lung OprF-specific IgG and IgA and increased lung T-cell OprF-specific INF-γ compared to immunisation with Ad5OprF. In general, the favourable mucosal immune responses in mice following immunisation with the AdC7 vaccine vector favours the further development of Ad vectors based on non-human primates as vaccines to induce protective lung mucosal immunity against *P. aeruginosa* [284].

Finally, the use of live attenuated pathogens such as *Salmonella* or *Francisella tularensis* as antigen delivery systems stand out among the attempts to improve the mucosal vaccine response to *P. aeruginosa* with mixed results. In general nasal and oral vaccination with attenuated *Salmonella* mutant strains induce a mixed response which is biased towards a Th1 response [329]. Preclinical studies [330,331] and a phase I-II clinical trial [332] involving *Salmonella* species for the nasal or oral delivery of *Pseudomonas* OprF or OprI, induced IgG and IgA antibodies. In contrast, in murine models, subcutaneous vaccination with an S. Typhimurium LH430 expressing OprF-OprI achieved the highest levels of protection and specific immunoglobulin titres than oral immunisation [333]. Importantly, a recent study, where an attenuated S. Typhimurium expressing *S. enterica* type III secretion effector protein, SseJ, and the *P. aeruginosa* PcrV elicited detectable levels of antigen-specific IgG in all mice, reduced bacterial loads in the spleens and lungs and the levels of proinflammatory cytokines (TNF-α and IL-6) and most of the vaccinated mice survived. In contrast, *Salmonella* expressing SseJ-OprF/I was not able to induce a specific IgG response and did not show significant protection against *P. aeruginosa* challenge [334]. Other groups have also used attenuated *Salmonella* for the delivery of polysaccharide antigens, such as PA103 serogroup O11 *O*-antigen [335,336,337,338]. *F. tularensis* has also been used for the delivery of *P. aeruginosa* PilA, OprF, and FliC. Mice immunised with LVS expressing *FliC_Pa_*, produced high levels of antibodies specific for *P. aeruginosa*, demonstrating the potential of this bacterial strain for the delivery of P. aeruginosa antigens [339].

## 6. Lessons from *P. aeruginosa* Vaccines and Future Directions

Over 50 years of research into the development of a vaccine against *Pseudomonas* has not yet yielded a marketable product. However, many lessons can be drawn which may improve future efforts. Most of the *P. aeruginosa* vaccine candidates developed to date were based on conventional protection mechanisms, i.e., opsonophagocytic antibody-mediated killing and/or antibody-mediated toxin inhibition [92]. However, these mechanisms may not be sufficient for protection. Considering that over 90% of pathogens access the body through mucosal sites, the use of mucosal vaccination should overcome the limitations of current vaccines to provide first-line protection against respiratory pathogens invasion and spread [344]. *P. aeruginosa* respiratory infections occur via interactions between the pathogen and the mucosa of the respiratory tract, so achieving mucosal immunity should be a goal for an effective vaccine

On the whole, Th2 cell responses do not appear to be protective against *P. aeruginosa* infections, so vaccines that elicit such responses may not be optimal. Th17 cell responses stand out as being key to protection against *P. aeruginosa* respiratory infections since they can increase mucosal immunity, as demonstrated with Th17 response-inducing adjuvants [92,94,137,323]. However, there are contradictions regarding the benefits of this type of cellular response in chronic infections, such as in CF people, and further studies are needed in this area. In addition, Th1 responses may contribute to improving the immune response against acute *Pseudomonas* infections, as suggested by the use of alum+NLX, and, interestingly, with the use of non-human adenovirus vectors in chronic infections [137,309]. Moreover, the use of live-attenuated bacteria such as *Salmonella* or *F. tularensis* is an interesting strategy for achieving mucosal immunity. While these studies give us hints, it remains unclear what the most effective immune response to combat *P. aeruginosa* is. A deeper understanding of host-pathogen interactions will facilitate the development of antigen–adjuvant systems capable of eliciting both cellular and humoral responses which work synergistically to give robust protective immune responses against *P. aeruginosa* infections.

The effectiveness of any vaccine depends on the correct selection of the antigen and adjuvant. Protein antigens show the most potential as they allow the identification of most protective epitopes, also facilitating the manufacture and optimisation of synthetic peptides and chimeric proteins. They can be easily and safely produced with relatively inexpensive large-scale purification. However, protein antigen variability and loss of expression during chronic infection is a challenge. Consequently, OMPs, which are surface exposed and far more conserved than other virulence factors, may result in broader coverage and better protection. Periplasmic proteins have greater solubility and are easier to purify, and their essential functions suggest that they will be conserved across serotypes, hence offering a broader coverage; however, further investigation of their potential in human trials is required.

Although a multitude of bacterial components showed potent immunogenic properties, none of them alone conferred the expected benefit in the clinical setting. Therefore, vaccines that present multiple antigenic components may be a more effective strategy, with the potential to induce a more complete and effective response. DNA vaccines, which have shown good survival rates in general, offer the possibility to simultaneously target different antigens. They can be easily prepared and harvested in vast quantities. However, it is a newly developed field, needing further evaluation in clinical studies. Eventually, the use of a multivalent vaccine that combines accessible and conserved antigens may ensure broad coverage and clinical efficacy.

The lack of a licensed vaccine against *P. aeruginosa* despite many years of effort is clear evidence of the great challenge involved and the need for further progress in understanding the complex *Pseudomonas*-host relationship at each stage of infection. The adaptability of *P. aeruginosa* and the large arsenal of virulence factors further complicate the realisation of a vaccine; however, the emergence of antibiotic multi-resistant strains and the susceptibility of vulnerable groups to *Pseudomonas* infections drives us in our quest to find effective antigen–adjuvant systems for the generation of a vaccine against this challenging pathogen.

## Figures and Tables

**Table 1:** Summary of pre-clinical studies on *P. aeruginosa* vaccine candidates elaborated.

Ag	Composition	Animal Model	Strains	Adjuvant	Administration Route; Dose	% Survival and Time *; Immune Response	Ref.
Lipopolysaccharide	LPS extracts from 16 strains (PEV-01)	Mice	OPS 1-16	None	IP; NS	NS	[112]
	Octavalent OPS-ETA conjugate (Aerugen^®^)	Rabbits, mice	ATCC 27316, PA53	AI(OH)_3_, CT, CpG1826	IM, IN; 2–50 µg	Neutralising, opsonic IgG, INF-γ, TNF-α	[113,114,115]
	Liposomes with core LPS	Rabbits	PAC608	AI(OH)_3_	IM; NS	70–88%, 48 h; cross-reactive Ab	[116]
Alginate	MEP extracts	Rats, mice, GP	Various	Muramyl dipeptide	IV, IM; 10–100 µg	33–50%, 4 days; opsonic Ab, IC	[117,118,119]
	MEP-ETA conjugate	Mice, rabbits	3064, PA103	AI(OH)_3_	IM; 10–50 µg	Anti-ETA opsonic Ab, anti-MEP IgG	[120]
	MEP-KHL conjugate	Mice, rabbits	2129	None, Freund’s	SC; 10 µg	Opsonic IgG	[121]
Flagellum	Monovalent FliC	Mice	Various	None	IM; 7–10 µg	40–100%, 5–10 days;	[122,123]
	Whole purified flagella	Mice	Various	None	IN; 2 µg	83,3%, 125 h; protective Ab	[124]
	r-flagellins	Mice	PAK, PAO1, BI1, BI2	Alum, Montanide ISA 70	SC, ID; 10–20 µg	25–90%, 7 days; IgG1, IL-4, INF-γ, IL-17, IL-12, IL-10	[125,126,127,128]
	FliC-MEP conjugate	Mice, rabbits	Various	Specol, Freund’s	SC, IN; 3–10 µg	30–85%, 5 days; opsonic IgG	[129,130]
	r-FliC-ETA chimeric protein	Mice	PAO1, 8821M	Freund’s	SC; 20 µg	80%, 10 days; IgG	[131]
	r-FliC-OMP chimeric protein	Mice, monkeys	PAK, PAO1, DM125, DM126	Freund’s, none	IM, SC; 1–50 µg	20–80%, 168 h; IgG, C3 complement	[132,133,134]
Type 4 pili	PilA C-terminus	Mice	PAK	Adjuvax™	IM; 50 µg	IgG1, IgG2	[135]
	Whole PilA	Mice	PAO1, clinical isolate	Freund’s, alum, naloxone	IT, SC; 0.004–5 µg	20–100% 72 h–7 days; IgA, IgG, IL-17	[136,137]
	PilA-ETA chimeric	Rabbits, mice	Various	Freund’s	SC, IN; 1–200 µg	Serum IgG, sIgA	[138,139]
	r- PilA and b-FliC	Mice	Various	None	SC; 15–20 µg	92–100%, 7 days; Th2 response, IgGs, IL-4, INF, IL-17	[140,141]
	PilA-PilQ chimeric	Mice	PAO1	Alum	SC; 10 µg	Opsonic IgG	[142,143]
	PilA-OPS conjugate	Mice	1244	None	IN, SC; 5 µg	40–65%, 225 h; anti-LPS IgG, IgM	[144]
	Consensus peptide	Mice	PAK, PAO1, KB7, P1	Adjuvax™	IP; 25–50 µg	20–40%, 48 h; cross-reactive Ab	[145,146]
	Low mw fimbrial pili	Mice	PAO1	Montanide ISA 70	IP; 50 µg	Th1/Th2 response	[147]
Outer membrane proteins	r-OprI	Mice	Various	AI(OH)_3_	IP; 25–27 µg	85–95.8%, 7–10 days; Ab	[148,149]
	r-OprF	Mice	Various	None	IM; 10 µg	36–92%, 10 days; Ab	[150]
	His-OprF-OprI protein (systemic formulation, IC43)	Mice	ATCC 33348, PAO1	AI(OH)_3_	IM; 20–100 µg	80% 7 days; IgG1, IL-3	[151,152]
	His-OprF-OprI protein (mucosal formulation)	Mice	ATCC 27853	AI(OH)_3_	IN; 30 µg	75%, 7 days; IgG, IgM, IgA	[153]
	OprF N-terminus	Mice	ATCC 9027	BCG, alum	SC; 12 µg	50%, 48 h; IgG1, IgG2a	[154]
	OprF-OprI-ETA chimeric protein	Mice	ATCC 15692, 33349, 29260	AlPO_4_	IP	60–80%, 10 days; neutralising Ab	[155]
	OprF, ETA and LasB	Rats	PAO1	AI(OH)_3_	IM; 25 µg	IgG	[156]
	OprF-OprI-PcrV chimeric protein	Mice	PAO1, PAK	Freund’s	SC; 10 µg	75%, 10 days; IgG	[157]
	OprI-OprF-AlgE PHA nanovaccine	Mice	PAO1	Alum	SC; 20 µg	Th1 response, IFN-γ, IgG2c	[158]
	r-OprL	Mice	Various	Freund’s, Curdlan	SC, IN; 50 µg	60–100%, 7 days; serum IgG, Th17 response	[159,160]
	r-OprH	Mice	PAO1, PA14, PA103	Curdlan	IN; 10 mg/mL	25–40%, 5 days; IgG	[161]
	r-OMP (PA5340, PA3526 and MotY)	Mice	PAO1	Alum	IP; 10 µg	20–50%, 5 days; NT	[6]
Type III secretion system	r-PcrV	Mice	PA103, PAO1, PAK	Freund’s, FCA-FIA, alum, CpG ODN	SC, IP, IN; 10 µg	29–90%, 1–7 days; IgG1, IgG2, IgA	[93,162,163,164]
	PcrV immunogenic epitopes	Mice	Various	Freund’s,	IM; 30 µg	50–65% 2–7 days; IgG, IL-17A, IFN-γ	[165,166]
	PcrV-C12HSL conjugate	Mice	PAO1	Freund’s	SC; 10–20 µg	75%, 14 days; IgG, IgM	[167]
	r-PcrV-OprI-Hcp1 chimeric protein	Mice	PAO1	Freund’s, MF59, Al(OH)_3_, AlPO_4,_ AS04	IM; 30 µg	80%, 7 days; IgG, IL-4, IFN-γ, IL-17A	[168]
	r-PopB-PcrH	Mice	PAO1	Curdlan	IN; 35 µg	62,5%, 5 days; IL-17	[92]
	PLGA encapsulated PopB-PcrH	Mice	PAO1	Curdlan	IN; 20 µg	70%, 6 days; IgG, Th17 response	[94]
Exotoxin A	r-ETA toxoid	Mice	Clinical mucoid strain	Freund’s, AlPO_4_	SC, IP; 1–20 µg	40–100%, 7 days; neutralising Ab	[169,170,171]
	PLGA encapsulated ETA	Mice	PAO1	None	IM; 100 µg	IgG, TNF-α, INF-γ, IL-4, IL-17	[172]
Elastase	LasB toxoid	Mice	IFO 3455	None	SC; 10 µg	NS	[173]
	LasB peptides-KLH/TT conjugate	Rats	PAO1, PAO2	Freund’s	IM, SC; 30 µg	IgG, IgA	[174]
Catalases	Purified KatA and KatE	Rats	385, 423	Freund’s	IPP; 10 µg	IgG, IgM, IgA, phagocytic cells	[175,176,177]
Auto-inducers	Hapten C12HSL-BSA conjugate	Mice	PAO1	Freund’s	SC; NS	36%, 7 days; serum Ab	[178]
Iron uptake proteins	r-FpvA, FoxA and HasR	Mice	PAO1, PA14, PA103	Curdlan	20 mg/mL	10–35%, 5 days; NS	[161]
	FpvA peptide-KLH conjugate	Mice	PAO1	Curdlan	IN; 35 µg	IgA, IgG, IgM, IL-17	[179]
	r-HitA	Mice	ATCC 9027	BCG, Freund’s, IFA	SC or IM; 15 µg	IgG	[180]
Whole-cell killed	Killed PA (Pseudostat^®^)	Rats	CF385	Freund’s	IPP, SC, IT; 10^10^ CFU/mL	PMN cells, IgA, IgG, IgM, TNF-α	[181,182,183]
	H_2_O_2_-deactivated PA	Mice	PAO1	None	3 × 10^7^ CFU/mL	100%, 6 days; IgG, IgM	[184]
	KBMA+IPTG, OSTAB pEi strain	Mice	CHA	None	SC; 1–2 × 10^8^ CFU/mL	58.3%, 150h; Ab, IL-17, Th17 cells,	[185]
Live-attenuated bacteria	PAO1Δ*aroA* and PA14Δ*aroA*	Mice, rabbits	Clinical isolates and lab strains	None	IN; 1 × 10^8^–2 × 10^9^ CFU/mL	40–100%, 7 days; IgG, neutrophils, IL-17	[186,187,188]
	O2/O5, O6, O10, and O11 serogroups *aroA* mutants	Mice	Various	None	IN; 1 × 10^8^–1 × 10^9^ CFU/mL	10–35%, 7 days; opsonic Ab, lung CD4 T cells	[189]
	PAO1Δ*murI*	Mice	PAO1, PA14, ST235	None	IN, IM; 3 × 10^7^–2 × 10^8^ CFU/mL	29–100%, 15 h; IgM, IgG, CD4+ T-cells	[190]
DNA vaccines	Type-a/-b flagellin	Mice	PAO1, PAK	None	IM; 50 µg	20–90%, 10 days; IgG	[191]
	OMPs	Mice, chickens	Various	None, Al(OH)_3_	IM, IP; 1–200 µg	40–93.3%, 8 days-6 weeks; opsonic IgG1, IFN-γ, IL-2	[192,193,194,195]
	OprF2-VP22	Mice	PAO1	None	IM; 20 µg	0–40%, 10 days; IgG1, IgG2a	[196]
	PilA, OprF-OprI and PcrV	Mice	PAO1, PAK, D4	None	IM, 100 µg	30–100%, 10 days; IgG	[197]

Abbreviations; Ab: antibody, Ag: antigen, CT: cholera toxin GP: guinea pig; ID: intradermal, IG: intragastrical, IM: intramuscular, IN: intranasal, IP: intraperitoneal, IPP: intestinal Peyer’s patch, IT: intratracheal, IV: intravenous, mw: molecular weight, NS: not specified, PA: *P. aeruginosa,* r-: recombinant, SC: subcutaneous. * Survival data as a percentage of immunised mice and time when reported.

**Table 2:** Summary of clinical studies on *P. aeruginosa* vaccine candidates elaborated.

Ag	Composition	Phase	No. of Patients (Type)	Administration Route; Dose	Adjuvant	Outcomes	Ref
**Lipopolysaccharide**	LPS extracts from seven strains (Pseudogen^®^)	II	12 (CF, chronic)	IM; 6–12 µg/Kg	None	Lack of clinical benefit, toxicity in 92% of patients	[198,199]
	LPS extracts from 16 strains (PEV-01)	I	15	SC; 0.5 mL	None	Appearance of protective antibodies	[200]
		II	34 (CF, non-PA colonisation)	SC; 0.25–0.5 mL	None	No reduction in colonisation, rapid clinical deterioration	[201]
	Octavalent OPS-ETA conjugate (Aerugen^®^)	I	20	SC; 0.5 mL (162.5 µg)	None	Safe, neutralising anti-ETA and opsonising anti-LPS IgG	[202,203]
		II	26 (CF paediatric)	IM; 6–12 µg/Kg	None	Only 35% colonisation, better lung preservation	[204]
		III	476 (CF)	NS	NS	No clinical difference between groups	[205]
**Alginate**	Purified MEP extracts	I	28	IM, SC; 0.5 mL	None	Poor production of opsonic Ab	[206]
**Flagellum**	Monovalent FliC	I	220	IM; 40 µg	Al(OH)_3_	IgG and IgA in serum and SIS	[207]
		II	10	IM; 40 µg	None		[208]
	Bivalent FliC	III	483 (CF non-PA infected)	IM; 40 µg	Al(OH)_3_, thiomersal	High serum IgG titres to flagella vaccine subtypes, 34% protection	[209]
**Outer membrane proteins**	OMP extractions from PA strains (CFC-101)	I	48	SC, IM; 0.1–1 mg	None	Safe, Immunogenic, TNF-α	[210]
		II	48 (burn)	IM; 0.5–1.0 mg	None	No clinical benefit	[211]
		II	95 (burn)	IM; 0.5–1 mg	None	Adverse reactions	[212]
	r-OprI	I	28	IM; 20–500 µg	None	High variability among volunteers	[213]
	His-OprF-OprI protein (systemic formulation, IC43)	I	32	IM; 20–500 µg	Al(OH)_3_	Increased IgG1 titres	[214]
		I	8 (burn)	IM; 100 µg	Al(OH)_3_	Well tolerated, increased Ab titres	[215]
		I	163	IM; 50–200 µg	Al(OH)_3_	Safe, specific IgG response	[216]
		II	137 (ventilated ICU)	IM; 100–200 µg	Al(OH)_3_	IgG response, lower mortality but higher infection in vaccinated group	[217]
		II-III	799 (medically ill ICU)	IM; 100 µg	None	No difference in mortality and survival rates	[9]
	His-OprF-OprI protein (mucosal formulation, SDS-emugel)	I	8	IN; 500 µg	None	Safe, increased IgG and IgA in 6 subjects over 3 months, inter-subject variability	[218]
		I-II	12	IN, systemic; 1 mg	None	Lasting immunity (IgG and IgA) in serum and airways	[219]
**Whole-cell killed**	Killed PA (Pseudostat^®^)	I	9 (bronchiectasis)	Oral, NS	NS	Safe	[220]
		I	30	Oral, 150 mg	None	Opsonic Ab, macrophage killing, few adverse events	[221]

Abbreviations; Ab: antibody, Ag: antigen, his-: His-tagged, ICU: intensive care unit, IM: intramuscular, IN: intranasal, NS: non-specified, PA: *P. aeruginosa*, r-: recombinant, SC: subcutaneous, SIS: secretory immune system.

**Table 3:** Examples of adjuvants used in *P. aeruginosa* vaccine candidates, the responses achieved against *P. aeruginosa* and their licensing status.

Adjuvant	Class	Mechanism or Receptor	IR against PA	Use in Humans	Ref
CpG ODN	IMS	TLR9	Ab, Th1, CD8 + T cells	Used in the human HBV vaccine (HEPLISAV-B).	[163,313,324,325]
Aluminium salts	B	NALP3, ITAM, Ag delivery	Ab, Th2	Several licensed vaccines (HAB, HBV, DTP, HiB).	[217,313,314,316]
Curdlan	DS	Dectin-1; DC activation directs the differentiation of effector Th17 cells	Ab, Th17	FDA approved (1996) for use as a stabiliser or texturiser in foods. No evidence of any toxicity	[179,326,340]
PLGA	DS	Ag delivery	Ab, Th17	Registered safe by the U.S. FDA for clinical use.	[94,341]
dmLT	B	Stimulates IL-17 secretion and increases the transport of secretory IgA (sIgA)	Ab, Th1, Th17	Phase I and II of ETEC and EVAX vaccines (no adverse events reported).	[95,328]
AS04	B	TLR4	Ab, Th2	Used as part of a HBV vaccine (Fendrix, GSK) and an HPV vaccine (Cervarix, GSK).	[325]
MF59	DS	Immune cell recruitment	Ab, Th2	Currently licensed as part of a flu vaccine (Fluad*™*, Seqirus)	[316,319,325]
AdC7 vector	DS	Ag delivery to mucosal sites	Ab, Th1	Preclinical studies in mice against *P. aeruginosa*, Ebola virus and malaria in mice models.	[284,341]
CIA06 (alum + dLos)	IMS	TLR4 agonist derived	Ab, Th1, Th17, Th2	Preclinical studies in mice on the H1N1 pandemic influenza vaccine Greenflu-S^®^.	[323]
NLX + alum	IMS	Opioid receptor antagonists	Ab, Th1, Th17, Th2	Approved by the FDA as a prescription drug, used to reverse opioid-induced respiratory depression.	[137]
Live-attenuated bacteria (S. Typhimurium, *F. tularensis* LVS)	DS	Ag delivery to mucosal sites	Ab, Th2, Th1 (biased)	Retains toxicity in humans and animals but since 2008, oral live-attenuated Ty21a *(S.* Typhi) vaccines have been recommended for typhoid control.	[342,343]

Abbreviations; Ab: antibody, Ag: antigen, B: both, DS: delivery system, IMS: immunostimulatory, IR: immune response, PA: *Pseudomonas aeruginosa*.

**Table 4:** Examples of *P. aeruginosa* adjuvanted vaccine evaluated in pre-clinical studies.

Ag/Adj	Route	*P. aeruginosa* Strains	Immunisation (N°x dose, Ag, Adj)	TR	Ref
PopB+PcrH/ Curdlan	IN	ExoU^+^ PAO1 and attenuated PAO1Δ*aroA* (C+) (7 × 10^5^ CFU/mouse)	3 × 35 µg/dose, 10 mg/mL	Th17	[92]
PopB+PcrH/Curdlan	IN	ExoU^+^ PAO1 and live-attenuated PAO1Δ*aroA* (C^+^) (7 × 10^5^ CFU/mouse)	3 × 35 µg/dose, 10 mg/mL	Th17	[94]
OMPs/dmLT	ID	PA01 (1.4 × 10^7^ CFU/mouse)	3 × 1 µg/mouse, 1 µg	Th1/17	[95]
OMP/CIA06	IM	GN-H3 and PA103 (10 LD_50_)	2 × 5 µg/mouse, 0.5 µg dLOS + 25 µg of alum in 100 µL of PBS	Th2/1/17	[323]
r-PilA/alum+NLX	SC	PA01 and a clinical isolate (3–5 × 10^6^ CFU)	3 × 5 µg/mouse, 6 mg/g body weight (NLX) and 200 mg of alum	Th2/Th1/Th17	[137]
PcrV/ FA, alum, or CpG ODN	IP	PA103 *exoS^-^, exoT^+^, exoU^+^ and exoY*^+^ (1.5 × 10^6^ CFU)	3 × 10 µg/dose, 100µL/dose FCA/FIA, 100 µL/dose alum or 10µg/dose of CpG ODN	FA and CpgODN: Th1/Th2. Alum: Th2	[93]
PcrV/CpG ODN	IN	PA103 strain (ATCC 29260, 1.5 × 10^6^ CFU in 60µL)	3 × 5 µg/mouse, 6 mg/g body weight and 200 mg of alum	Th2/Th1	[163]
OprF/AdC7 (AdC7OprF)	IM, IT	PA encapsulated in agar beads (5 × 10^6^ CFU, 50μL).	1 × 10^9^–10^11^ pu/animal	Th2/Th1	[309]
SseJ-PcrV and SseJ-OprF-I/Attenauated S. Typhimurium SV9699	IP	PAO1 (9 × 10^6^ bacteria in 0.2 mL)	1 × 2 × 10^5^ CFU/animal	NS	[334]
PilA*_Pa_*, OprF*_Pa_*, FliC*_Pa_*/Attenuated *F. tularensis* LVS	IN	PA1244 (PilA*_Pa_*) and PA14 (OprF*_Pa_*, and FliC*_Pa_*)	1 × ~100 CFU/animal	NS	[339]

Abbreviations; Adj: adjuvant, Ag: antigen, ID: intradermal, IM: intramuscular IN: intranasal, IP: intraperitoneal, IT: intratracheal, NS: not specified, SC: subcutaneous, TR: type of response.
